# Identification of substrates and sequence requirements for CARNMT1-mediated histidine methylation of C3H zinc fingers

**DOI:** 10.1016/j.jbc.2025.110335

**Published:** 2025-06-03

**Authors:** Jędrzej M. Małecki, Sara Weirich, Manuel Ramirez-Garrastacho, Lars Hagen, Jakin Al-Egly, Jan H. Anonsen, Lisa Schroer, Maria C. Herrera, Erna Davydova, Geir Slupphaug, Albert Jeltsch, Pål Ø. Falnes

**Affiliations:** 1 Department of Biosciences, Faculty of Mathematics and Natural Sciences, University of Oslo, Oslo, Norway; 2 CRESCO - Centre for Embryology and Healthy Development, University of Oslo and Oslo University Hospital, Oslo, Norway; 3 Department of Molecular Biochemistry, Institute of Biochemistry, University of Stuttgart, Stuttgart, Germany; 4 Department of Clinical and Molecular Medicine, Faculty of Medicine and Health, Norwegian University of Science and Technology, Trondheim, Norway; 5 The Proteomics and Modomics Core Facility, PROMEC, at NTNU and the Central Norway Regional Health Authority, Stjørdal, Norway

**Keywords:** methylation, protein histidine methyltransferase, carnosine, 1MH, πMH, MYLK2, C3H zinc finger, U2AF1, RNF113A, spliceosome, peptide array, mass spectrometry, substrate specificity, post-translational modification

## Abstract

It has recently become clear that protein histidine methylation is widespread and functionally important in many cellular processes, and human CARNMT1 was recently reported as a novel protein histidine methyltransferase (HMT). We describe our independent uncovering of CARNMT1's protein HMT activity and a comprehensive assessment of its methylation targets and substrate specificity. Using a combination of *in vitro* methylation of cellular extracts and protein mass spectrometry, we identified several CARNMT1 substrates that were fully methylated in cells, all of which were C3H zinc finger (ZnF) proteins. These include the previously identified U2AF1, ZC3H15, and ZC3H18 but also the unreported RBM22, PPP1R10, PRR3, and RNF113A. Using peptide arrays, we investigated CARNMT1-mediated methylation of 145 candidate sequences, encompassing all C3H ZnFs and selected non-ZnFs. We found that only ∼30% of the tested sequences were methylated, with C3H ZnFs constituting the vast majority of the strongly methylated ones, most of which are also methylated in cells. This establishes peptide methylation as a good predictor of *in vivo* methylation. To investigate the specificity of CARNMT1, we systematically substituted His-proximal residues in four different substrate peptides. This generated four rather different sequence preference profiles, which were still quite restrictive for each peptide, indicating that substrate sequence recognition by CARNMT1 is context-dependent and that sequence-based prediction of additional CARNMT1 substrates may be challenging. We also identified several homologous methylation events in *Caenorhabditis elegans* and showed that they could be introduced by nematode CARNMT *in vitro*. Thus, CARNMT1 is an evolutionarily conserved protein HMT with a complex mode of substrate recognition.

The human genome encodes approximately 200 *S*-adenosyl-L-methionine (AdoMet)-dependent methyltransferases (MTases) categorized in different groups (1). The largest group consists of the seven-β-strand (7BS) MTases, characterized by a core fold of seven β-strands, which collectively methylate a wide range of substrates, including small molecules, nucleic acids, and proteins (2, 3). The second largest group comprises the SET-domain proteins, best known for lysine methylation at the flexible tails of histone proteins (4). Although certain biochemical functions of many human MTases have been uncovered, some of these enzymes remain either uncharacterized or incompletely described, and numerous cellular methylation events are yet to be assigned to specific MTases.

Many proteins are subject to methylation, particularly at lysines and arginines, but other residues such as glutamine and histidine, as well as the N- and C-termini, can also be methylated (5, 6, 7, 8, 9). Histidine can be monomethylated at one of two nitrogen atoms of the imidazole ring (denoted N1 and N3, or π and τ, respectively), yielding either 1-methylhistidine (1MH, πMH) or 3-methylhistidine (3MH, τMH) (8, 10). Methylation increases the bulkiness of the imidazole ring and changes its hydrogen-bonding properties and pK_a_ value. So far, two 3MH-specific protein histidine MTases (HMTs) have been described in humans, namely, SETD3 (11, 12) and METTL18 (13, 14), which methylate actin and the ribosomal protein RPL3, respectively. The first identified 1MH-specific protein HMT, METTL9, targets several proteins at H-X-H motifs, for example, the antimicrobial and immunomodulatory protein S100A9, the mitochondrial respiratory Complex I subunit NDUFB3, and various zinc transporter proteins (15, 16, 17). Recent protein mass spectrometry (MS) data reported the existence of hundreds of histidine-methylated sites across numerous proteins, indicating that this modification is common and widespread (12, 18, 19). For many of these histidine methylation events, the identity of the responsible protein HMT remains elusive.

Carnosine methyltransferase 1 (CARNMT1) was originally characterized in mammals as an MTase introducing 1MH in the dipeptide carnosine (β-alanyl-histidine), thereby converting it to anserine (20). Carnosine and anserine are abundant in muscles and the brain, but their exact physiological roles in these organs remain somewhat unclear (21). Since putative CARNMT1 orthologs are also found in yeast and plants, which lack carnosine, it was suggested that this MTase may in fact introduce 1MH in proteins as well (20). Indeed, two independent groups recently reported CARNMT1-mediated methylation of His residues present in C3H-type zinc finger (C3H ZnF) domains (22, 23), thereby influencing mRNA splicing and turnover.

In the present study, we report our independent discovery of CARNMT1 as a protein HMT, as well as the results of an extensive investigation of its substrate repertoire and enzymatic specificity. We show that recombinant CARNMT1 methylates multiple proteins in cytosolic and nuclear extracts of human *CARNMT1* KO cells. Using MS, we detected stoichiometric CARNMT1-dependent histidine methylation of several proteins within their C3H ZnF domains. These include the previously discovered CARNMT1 substrates U2AF1, ZC3H15 and ZC3H18 as well as the novel targets RBM22, PPP1R10, PRR3 and RNF113A. To further explore CARNMT1-mediated methylation, we analyzed a comprehensive set of 119 human C3H ZnF-derived sequences using peptide arrays, and identified 30 methylation targets, 22 of which showed strong methylation. In addition, we tested methylation of 26 non-ZnF sequences reported as histidine-methylated *in vivo* and found that 10 of them were methylated by CARNMT1, though only 2 exhibited strong methylation. Taken together, our *in vitro* and *in vivo* methylation data indicate that C3H ZnFs represent the primary protein substrates of CARNMT1, but only a minority of the human C3H ZnFs are actually subject to CARNMT1-mediated methylation. To investigate the specificity of CARNMT1, we systematically substituted the His-proximal residues in four different substrate peptides. This generated four rather different sequence preference profiles, which were still quite restrictive for each peptide, indicating that the overall peptide sequence, in a complex manner, modulates the recognition by CARNMT1 of residues at specific positions in the substrate. We also identified several homologous methylation events in *Caenorhabditis elegans* and showed that they could be introduced by the nematode CARNMT *in vitro*. In summary, our study indicates that CARNMT1 is an evolutionary conserved protein HMT with a complex mode of substrate recognition and uncovers several novel protein substrates of this enzyme.

## Results

### Human CARNMT1 is a histidine-specific MTase introducing 1MH in MYLK2

**Figure 1:**
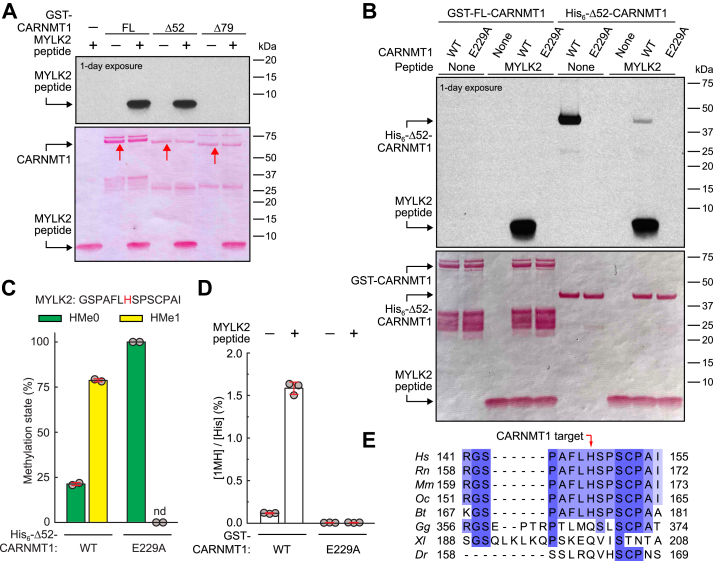
**Human CARNMT1 introduces 1MH in MYLK2.***A*, CARNMT1-dependent methylation of a candidate MYLK2-derived peptide substrate *in vitro*. 5 μg of synthetic MYLK2-derived peptide (aa. 141–155) was incubated with [^3^H]-AdoMet (0.5 μCi) and 1.5 μg of recombinant GST-tagged CARNMT1, either full-length (FL), Δ52 or Δ79, and then separated by SDS-PAGE and transferred to a membrane. Incorporation of [^3^H]-methyl groups into the peptide was visualized by fluorography (*top*) of a Ponceau S-stained membrane (*bottom*). *Red* arrows indicate the position of various GST-CARNMT1 variants. *B*, the E229A mutation of CARNMT1 abrogates its enzymatic activity. MYLK2 peptide was incubated with [^3^H]-AdoMet and GST-tagged FL-CARNMT1 or His-tagged Δ52-CARNMT1, either WT or E229A-mutated, and [^3^H]-methyl incorporation into peptide and proteins was visualized by fluorography as in (A). *A, B*, shown are representative images from one of three independent experiments. *C*, the MYLK2 peptide is monomethylated by CARNMT1 at a His residue. 0.02 μg MYLK2 peptide was incubated with unlabeled AdoMet (1 mM) and 1 μg His-tagged Δ52-CARNMT1, either WT or E229A-mutated. Samples were acetone-precipitated, trypsin-digested and analyzed by MS. Shown are the mean relative intensities of MS signals, gated for unmethylated (HMe0) and monomethylated (HMe1) states of the indicated peptide, with the targeted His marked in *red*. Examples of MS/MS spectra of identified peptides are provided in Supporting Information 2. Error bars represent standard deviation (SD) of two independent experiments. nd: not detected. *D*, CARNMT1 introduces 1MH modification in the MYLK2 peptide. 0.2 μg of MYLK2 peptide was incubated with AdoMet (1 mM) and 1.5 μg GST-tagged CARNMT1, either WT or E229A-mutated. Samples were subjected to HCl-mediated hydrolysis into amino acids, and the content of 1MH, 3MH and His were determined by LC-MS/MS. Shown is the average amount of detected 1MH, expressed as percentage of total His. 3MH was detected at background levels. Error bars represent SD of three independent analyses. *E*, H148 in MYLK2 is conserved only in mammals. An alignment of MYLK2 homologs from various organisms was generated applying the MAFFT algorithm embedded in Jalview v.2 on RefSeq sequences of putative MYLK2 homologs from *Homo sapiens* (Hs, NP_149109.1), *Rattus norvegicus* (Rn, NP_476557.2), *Mus musculus* (Mm, NP_001074513.2), *Oryctolagus cuniculus* (Oc, NP_001075705.1), *Bos taurus* (Bt, NP_001077188.1), *Gallus gallus* (Gg, NP_990723.2), *Xenopus laevis* (Xl, XP_041433382.1) and *Danio rerio* (Dr, NP_001103990.1). A *red arrow* indicates the position of H148 in human MYLK2.

Until recently, myosin light chain kinase 2 (MYLK2) from rabbit skeletal muscles was one of the few proteins reported to contain 1MH (24). Since CARNMT1 was a candidate 1MH-specific protein HMT, we decided to explore whether it could methylate a peptide encompassing the known methylation site, H148, in human MYLK2. To test this, we incubated GST-tagged recombinant CARNMT1 with a human MYLK2-derived peptide (residues 141–155) in the presence of [^3^H]-AdoMet and detected methylation as a radiolabeled band after SDS-PAGE and fluorography. We tested full-length GST-CARNMT1 (NP_689633.1) as well as two N-terminally truncated human CARNMT1 variants: Δ79 (an isoform lacking the first 79 amino acids; NP_001307426.1) and Δ52 (lacking the first 52 amino acids and matching the size of the CARNMT1 homolog from *Saccharomyces cerevisiae*) (Fig. S1). We observed a strong [^3^H]-labeling of a single band corresponding to the MYLK2-derived peptide, suggesting that CARNMT1 is the protein HMT responsible for MYLK2 histidine methylation (Fig. 1*A*). This was only observed with the full-length and Δ52-CARNMT1 but not with the Δ79 truncation variant, which appears to be catalytically inactive. We also generated a His-tagged version of Δ52-CARNMT1, which was easier to express and purify, but displayed strong auto-methylation activity, likely due to methylation of its His-tag (Fig. 1*B*). The His-tagged protein was used selectively in experiments where auto-methylation did not interfere with the results, while GST-tagged CARNMT1 was used in other assays. Importantly, the MYLK2-derived peptide was not methylated by the catalytically inactive E229A mutant (Fig. 1*B*), where a glutamine residue predicted to be crucial for AdoMet binding, based on its position in the conserved 7BS MTase motif Post I (25), was mutated (Fig. S1). This rules out the possibility that the observed [^3^H]-labeling resulted from a contaminating, co-purifying *Escherichia coli*-derived MTase.

Mass spectrometry analysis of the MYLK2-derived peptide incubated with unlabeled AdoMet and His-tagged CARNMT1 revealed efficient monomethylation at the His residue corresponding to H148 in the MYLK2 protein (Fig. 1*C*; Supporting Information 2 shows MS/MS spectra). Amino acid analysis of products from a reaction containing the MYLK2-derived peptide, GST-CARNMT1, and AdoMet showed that CARNMT1 generates 1MH (Fig. 1*D*). Reassuringly, in both assays, histidine methylation was absent when the inactive E229A mutant was used as a negative control.

We also set out to investigate whether CARNMT1 could methylate recombinant full-length MYLK2, but our efforts to express this protein were unsuccessful. However, we generated a recombinant GST-tagged MYLK2 fragment consisting of amino acids 1 to 261, *i.e.,* the N-terminal, unstructured domain of MYLK2 that lacks the C-terminal kinase domain. The purified GST-MYLK2 (1–261) appeared as two major bands of ∼50 kDa, where the upper band corresponds to the expected size of the protein (53.4 kDa). We observed efficient methylation only of the lower band, and this was abolished by introducing the H148A mutation (Fig. S2), indicating that methylation occurs also in the context of the recombinant protein, and that H148 is the sole methylation site in this MYLK2 fragment.

In summary, these experiments establish human CARNMT1 as an active protein HMT that introduces 1MH at H148 in MYLK2 *in vitro*. Notably, a sequence alignment of vertebrate MYLK2 homologs revealed that H148 is present only in mammals (Fig. 1*E*), indicating that MYLK2 represents a recently evolved CARNMT1 substrate. However, given the widespread distribution of putative CARNMT1 orthologues across different species, one may speculate that CARNMT1 also targets other, more evolutionary conserved protein substrates.

### CARNMT1 introduces 1MH in a large portion of the cellular proteome

**Figure 2:**
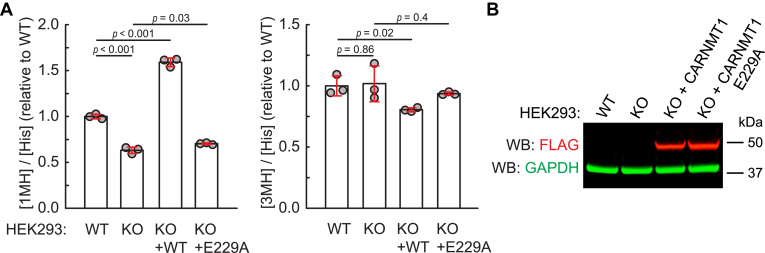
**CARNMT1 is responsible for a large portion of 1MH present in proteins from HEK293 TRex cells.***A*, *CARNMT1* KO cells show a reduction in 1MH content. WT or *CARNMT1* KO cells, or KO cells expressing WT or E229A-mutated CARNMT1-FLAG, were lysed, and proteins precipitated with 10% TCA were subjected to HCl-mediated hydrolysis into amino acids. The content of 1MH, 3MH and His was determined by LC-MS/MS. Shown is the detected amount of 1MH and 3MH (relative to total His) relative to that of WT cells. Error bars represent SD of three independent analyses. *p*-values were calculated using Student’s *t* test. *B*, expression of CARNMT1-encoding constructs in complemented cells. *CARNMT1* KO cells were complemented with CARNMT1-FLAG, either WT or E229A-mutated, and treated for 24 h with doxycycline (1 μg/ml), to induce expression of CARNMT1-FLAG, which was detected by western blotting using anti-FLAG antibody. Glyceraldehyde phosphate dehydrogenase (GAPDH) is included as a loading control. Non-complemented WT and *CARNMT1* KO cells are included as negative controls.

Next, we set out to investigate how much of the 1MH in the human proteome is attributable to CARNMT1. To assess this, we compared, using LC-MS/MS, the 1MH content in WT and *CARNMT1* KO human HEK293-derived TRex cells. We found that the 1MH/His ratio was reduced by ∼40% in KO compared to WT cells, whereas the 3MH/His ratio remained unchanged (Fig. 2*A*). Moreover, in KO cells complemented with WT CARNMT1-FLAG (Fig. 2*B*), the 1MH/His ratio was ∼60% higher than in WT cells, whereas cells complemented with the inactive E229A mutant showed a 1MH/His ratio similar to that of KO cells (Fig. 2*A*). These results, which are in line with similar data from Shimazu *et al*. (22), indicate that CARNMT1 is responsible for introducing 1MH in a large portion of the cellular proteome, but also suggest that the extent of CARNMT1-dependent methylation in WT cells is limited by enzyme availability.

### Identification of C3H ZnF proteins as CARNMT1 targets

**Figure 3:**
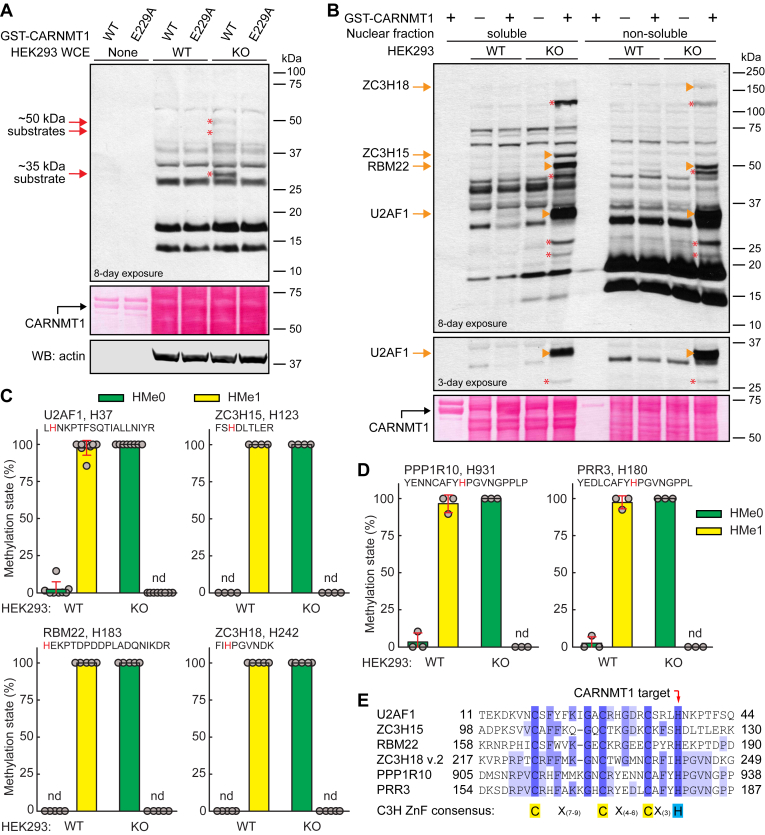
**Identification of C3H zinc finger (ZnF) proteins as CARNMT1 targets in HEK293 TRex cells.***A*, recombinant CARNMT1 methylates proteins in *CARNMT1* KO cell extracts. 50 μg protein from WT or *CARNMT1* KO whole-cell extracts (WCE) was incubated with [^3^H]-AdoMet (0.5 μCi) and 1.5 μg of recombinant GST-tagged CARNMT1, either WT or E229A-mutated, and then separated by SDS-PAGE and transferred to a membrane. Incorporation of [^3^H]-methyl groups into the proteins was visualized by fluorography (*top*) of a Ponceau S-stained membrane (*middle*). The membrane was probed with anti-actin antibody as a loading control (*bottom*). *Red* arrows and asterisks indicate positions of [^3^H]-labeled bands indicating putative CARNMT1 protein substrates. An extended version of this panel, including the results obtained with two additional, independent KO clones, can be found in Fig. S3*A*. *B*, recombinant CARNMT1 methylates proteins in the nuclear fraction of *CARNMT1* KO cells. *CARNMT1* KO cells were lysed and fractionated into cytoplasmic and nuclear fraction. 50 μg protein from the nuclear fraction was incubated with [^3^H]-AdoMet and GST-CARNMT1, and then subdivided into a soluble and non-soluble part by centrifugation. Protein methylation was assessed by fluorography as in (A). *Orange* arrows indicate [^3^H]-labeled bands in which U2AF1, ZC3H15, RBM22 and ZC3H18 were identified as CARNMT1-dependent histidine methylated proteins in a parallel MS analysis presented in (C). *Red* asterisks indicate unidentified CARNMT1 substrates. (A, B) Shown are representative images from one of three independent experiments. *C, D*, *CARNMT1* KO abrogates stoichiometric histidine methylation of several cellular proteins. WT and *CARNMT1* KO cells were lysed and fractionated into cytoplasmic and nuclear fraction, which was separated into a soluble and non-soluble part. *C*, fractions were either run on SDS-PAGE, and the regions of the gel corresponding to bands indicated in (B) and Fig. S3*B*, were cut out, and subjected to trypsin digestion and MS analysis. *D*, alternatively, proteins in the nuclear fraction were acetone-precipitated and subjected to trypsin digestion and MS analysis. *C* and *D*, shown are the mean relative intensities of MS signals, gated for unmethylated (HMe0) and monomethylated (HMe1) states of indicated peptides with the targeted histidine marked in *red*. Examples of MS/MS spectra of identified peptides are shown in Supporting Information 2. Error bars represent SD of three to eight independent experiments (some of the substrates in (C) were detected also in the acetone-precipitated material from (D)). nd: not detected. *E*, location of CARNMT1-targeted histidines (red arrow) within C3H ZnF sequences. A partial sequence alignment was generated for human U2AF1 (NP_006749.1), ZC3H15 (NP_060941.2), RBM22 (NP_060517.1) and ZC3H18 v.2 (NP_653205.3), PPP1R10 (NP_001363124.1) and PRR3 (NP_079539.2).

Given the likely existence of additional CARNMT1 substrates, we set out to identify these. To this end, we prepared whole-cell extracts (WCE) from HEK293 TRex WT and *CARNMT1* KO cells and incubated them with recombinant GST-CARNMT1 and [^3^H]-AdoMet. Using this approach, *in vitro* methylated proteins can be detected as radiolabeled bands after SDS-PAGE and fluorography. While CARNMT1 substrates will be unmethylated in extracts from KO cells, they may already be stoichiometrically methylated in WT cells and thus not be subject to further *in vitro* methylation. Therefore, high-occupancy methylation events will appear as bands with substantially stronger labeling in KO extracts compared to WT extracts. Using this approach, we observed labeling of putative CARNMT1 substrates with sizes of ∼35 and ∼50 kDa (Fig. 3*A* and S3*A*). Moreover, no methylation was observed with the inactive E229A mutant, ruling out the possibility that the observed activity was caused by an MTase already present in the extracts or co-purifying with recombinant CARNMT1.

To expand our substrate identification, we prepared cytoplasmic and nuclear fractions, which again were subfractionated into a soluble and non-soluble part. This revealed several bands with enhanced radiolabeling in the KO extracts (Fig. 3*B* and S3*B*), and we set out to identify the corresponding putative CARNMT1 substrates. To this end, we used protein mass spectrometry of tryptic digests of the cell extracts, or their fractionated counterparts, to identify histidine methylation events that were found in WT but were missing in KO cells. Proteins found to be methylated by CARNMT1 are summarized in Table 1, while Supporting Information 2 provides examples of MS/MS spectra and tables of detected y- and b-ions for unmethylated and His-methylated peptides detected in this study. For example, we identified H37 in the splicing factor U2AF 35 kDa subunit (U2AF1) as a CARNMT1 substrate present in the intense ∼35 kDa band (Fig. 3, *B* and *C*, and Fig. S3*B*). Similarly, we identified CARNMT1 methylation of H183 in the pre-mRNA splicing factor RBM22, H123 in the zinc finger C3H domain-containing protein 15 (ZC3H15), and H242 in the zinc finger C3H domain-containing protein 18 (ZC3H18), which are present in the ∼50, ∼55 and ∼150 kDa regions of the SDS-PAGE gels, respectively. Despite considerable efforts, we were not able to identify additional CARNMT1 substrates by MS analysis of specific bands. However, MS analysis of the total nuclear fraction, besides rediscovering some of the above-mentioned methylation events, revealed two additional CARNMT1 substrates: H931 in serine/threonine-protein phosphatase 1 regulatory subunit 10 (PPP1R10) and H180 in proline-rich protein 3 (PRR3) (Fig. 3*D*).

**Table 1:** CARNMT1 target sites identified in this study

Protein	C3H ZnF	Target site	Methylation of		
			Peptide^a^	Recombinant protein	Cellular protein^b^
MYLK2		H148	+++	yes^c^	
U2AF1	yes	H37	+++	yes	∼100%
ZC3H15	yes	H123	++		∼100%
RBM22	yes	H183	-		∼100%
ZC3H18	yes	H242	++		∼100%
PPP1R10	yes	H931	+		∼100%
PRR3	yes	H180	+		∼100%
RNF113A	yes	H221	+++	yes	∼95%
RNF113B	yes	H215	+++	yes	
ADAR		H21	+		
CDK20		H162	+		
CPSF4	yes	H59	++		
CPSF4L	yes	H59	+++		
	yes	H86	++		
	yes	H114	++		
DDIAS		H375	(+)		
DHX57	yes	H323	++		
DUS3L	yes	H145	+++		
ENO1		H133	+		
HELZ2	yes	H243	(+)		
MKRN1	yes	H79	+		
	yes	H108	++		
	yes	H232	+		
	yes	H390	++		
MKRN2		H13	++		
	yes	H26	++		
	yes	H55	++		
	yes	H189	+		
	yes	H347	+++		
		H389	(+)		
MYO1B		H791	+		
PITHD1		H208	+		
RC3H1	yes	H438	(+)		
SPEN		H1532	+		
ZC3H8	yes	H215	(+)		
	yes	H268	++		
ZC3H10	yes	H96	++		
ZFP36	yes	H128	++		
	yes	H166	++		
ZFP36L1	yes	H177	+++		
ZFP36L2	yes	H216	+++		

a Methylation intensity: +++, very strong; ++, strong; +, moderate; (+), weak; -, no methylation.

b Methylation status.

c N-terminal domain.

A sequence alignment and domain annotation of the identified CARNMT1 methylation sites in U2AF1, RBM22, ZC3H15, ZC3H18, PPP1R10, and PRR3 revealed that in each case the targeted histidine was part of a C3H ZnF domain (Fig. 3*E*). This is in agreement with the recent studies by Shimazu *et al.* and Wang *et al.* (22, 23), who reported CARNMT1-dependent methylation of histidines in C3H ZnFs, including the methylation of U2AF1, ZC3H15 and ZC3H18 described earlier. Our identification of RBM22, PPP1R10, and PRR3 as CARNMT1 substrates is unique to the present study. All CARNMT1 cellular targets identified in our study, as well as those reported by others, are summarized in Table 2.

**Table 2:** CARNMT1 target sites methylated in cells^d^

Protein	Target residue	C3H ZnF	Peptide methylation^a^	Methylation in cells^b^	Reference^c^
MYLK2	H148		+	∼3% (EN), ∼92% (OE)	(22)
U2AF1	H37	yes	+	∼100% (EN)	This study (22, 23),
ZC3H15	H123	yes	+	∼100% (EN)	This study (22, 23),
RBM22	H183	yes	-	∼100% (EN)	This study
ZC3H18	H242	yes	+	∼100% (EN)	This study (22, 23),
PPP1R10	H931	yes	+	∼100% (EN)	This study
PRR3	H180	yes	+	∼100% (EN)	This study
RNF113A	H221	yes	+	∼95% (EN)	This study
ZC3H8	H215	yes	+	∼100% (EN)	(23)
	H268	yes	+	∼45% (EN)	(23)
MKRN2	H26	yes	+	∼98% (EN)	(22)
	H55	yes	+	∼100% (EN)	(22)
	H71		-	∼37% (EN), ∼100% (OE)	(22)
	H189	yes	+	∼9% (EN), ∼100% (OE)	(22)
	H347	yes	+	∼4% (EN), ∼100% (OE)	(22)
	H389		+	0% (EN), ∼13% (OE)	(22)
	H407		-	1% (EN), ∼69% (OE)	(22)
MKRN1	H79	yes	+	∼98% (OE)	(22)
	H108	yes	+	∼98% (OE)	(22)
	H232	yes	+	∼33% (OE)	(22)
MBNL2 v2	H336		-	∼14% (OE)	(22)
MBNL3	H324		-	∼15% (OE)	(22)
CPSF4	H59	yes	+	∼100% (OE)	(22)
	H86	yes	-	∼25% (OE)	(22)
	H114	yes	-	∼21% (OE)	(22)
ZFP36	H23		-	∼9% (OE)	(22)
	H166	yes	+	∼29% (OE)	(22)
ZFP36L1	H177	yes	+	∼5% (OE)	(22)
PITHD1	H208		+	∼7% (OE)	(22)
RC3H1	H438	yes	+	∼30% (OE)	(22)

+, methylation; -, no methylation; EN, endogenous CARNMT1; OE, overexpressed CARNMT1.

a Detected in this study using peptide arrays.

b Methylation status.

c Reference for methylation in cells.

d With methylation level >5%.

### U2AF1 is methylated by CARNMT1 at H37 *in vitro*

**Figure 4:**
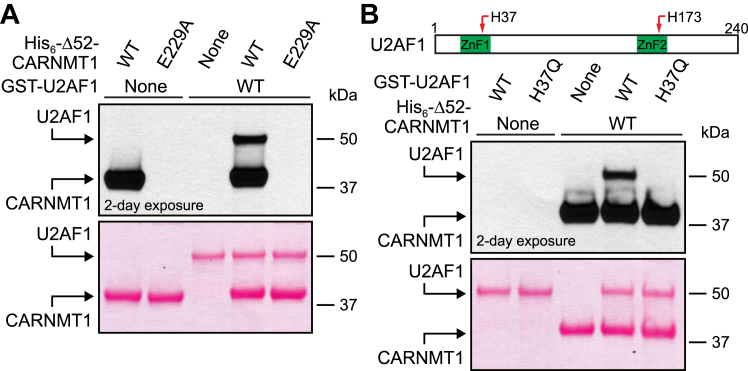
**Recombinant U2AF1 is methylated by CARNMT1 at H37.***A*, CARNMT1 methylates recombinant U2AF1. 0.5 μg of GST-tagged U2AF1 (aa. 1–199) was incubated with [^3^H]-AdoMet (0.5 μCi) and 1 μg recombinant His-tagged Δ52-CARNMT1, either WT or E229A-mutated, and then separated by SDS-PAGE and transferred to a membrane. Incorporation of [^3^H]-methyl groups into proteins was visualized by fluorography (*top*) of a Ponceau S-stained membrane (*bottom*). *B*, CARNMT1 methylates U2AF1 at H37. His-tagged Δ52-CARNMT1 was incubated with [^3^H]-AdoMet and GST-tagged U2AF1, either WT or H37Q-mutated, and protein methylation was assessed as in (A). *Upper panel*: diagram representing localization of H37 and H173 (*red arrows*) in C3H ZnF domains (*green*) of U2AF1. *A, B*, shown are representative images of one of three independent experiments.

The abovementioned results suggested that U2AF1 is a major cellular target of CARNMT1, and we therefore investigated whether recombinant U2AF1 is directly methylated by CARNMT1 *in vitro*. His-tagged CARNMT1 was incubated with [^3^H]-AdoMet and recombinant GST-tagged U2AF1 (aa. 1–199; we were unable to express the full-length 240 aa. protein). This revealed that U2AF1 was methylated by WT CARNMT1 but not by the inactive E229A mutant (Fig. 4*A*). U2AF1 has two C3H ZnFs, where ZnF1 contains the observed cellular methylation site H37, and ZnF2 contains H173, which we found in an unmethylated state in cells (Supporting Information 2). In agreement with these findings, the H37Q mutation abolished CARNMT1-mediated methylation of recombinant U2AF1 *in vitro*, indicating that H37 is the only target of CARNMT1 within the tested U2AF1 fragment (Fig. 4*B*).

### Identification of additional CARNMT1 targets using peptide arrays

**Figure 5:**
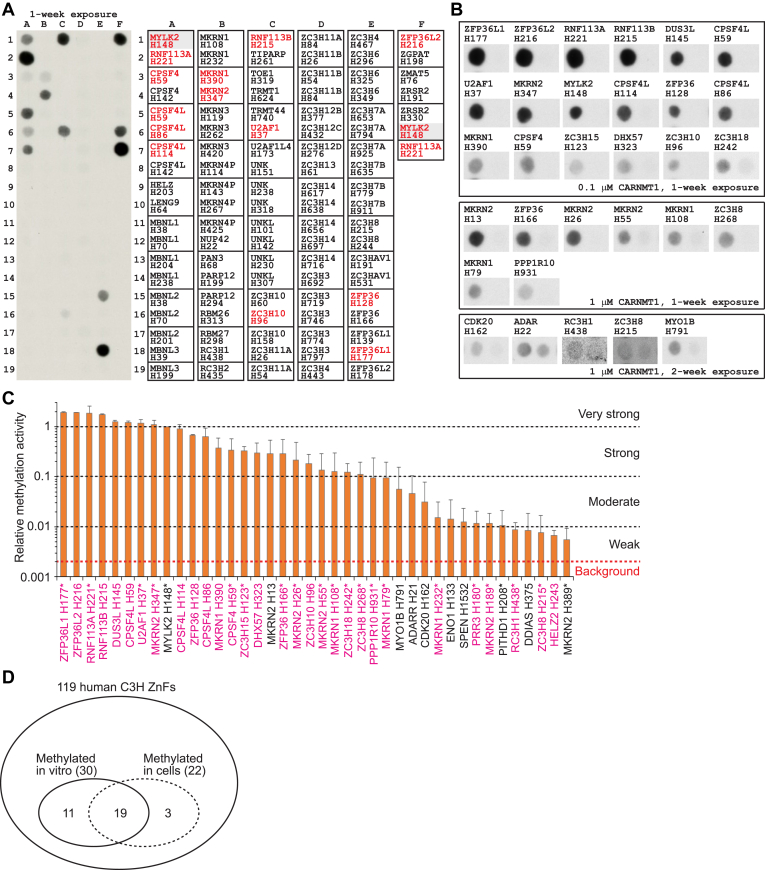
**Only a subset of human C3H ZnF sequences are methylated by CARNMT1 at the peptide level.***A*, methylation of human C3H ZnF-derived peptides by CARNMT1 *in vitro*. SPOT peptide arrays were prepared using 15-mer peptides derived from indicated proteins and containing indicated His at the center. The arrays were incubated with [^3^H]-AdoMet (0.5 mCi/ml) and recombinant His-tagged Δ52-CARNMT1 (0.1 μM). The incorporation of [^3^H]-methyl groups into peptides was then visualized by fluorography. Strongly methylated peptides are marked in *red*. Shown is an exemplary image of a peptide array, data were obtained in at least two independent replicates. See Supporting Information 3 for sequences of all tested peptides. *B*, methylation of human C3H ZnF-derived peptides and selected non-ZnF peptides by CARNMT1 *in vitro*. SPOT peptide arrays were prepared using 15-mer peptides derived from the indicated proteins and containing the indicated His at the center (*left*). Control peptides with His-to-Ala mutation of the central His were included as well (*right*). The array was incubated and processed as in (A), with indicated amount of recombinant CARNMT1. Shown are exemplary images of a peptide array, obtained in at least two independent replicates. *C*, summary of histidine-specific and CARNMT1-dependent methylation of indicated proteins and targeted histidines *in vitro*. SPOT arrays were prepared as in (B) and contained the MYLK2-derived peptide (H148) as a positive reference. Methylation intensities were calculated by subtracting the signal from His-to-Ala mutated peptides as background and are reported relative to the methylation intensity of the MYLK2 peptide, which is set at 1. The background level is set at 0.002, indicated by the *red* dashed line. Shown are average relative intensities calculated from 2 to 6 replicates with error bars representing standard deviation. Magenta indicates C3H ZnF-derived sequences, whereas non-ZnF sequences are in *black*. Asterisks indicate targets reported to be methylated by CARNMT1 in cells. *D*, diagram showing substantial overlap between the C3H ZnF sequences shown to be methylated by CARNMT1 *in vitro* and in cells. Note that the methylation status of the 119 non-redundant C3H ZnFs has not been systematically investigated in cells. Thus, the indicated number merely represents the CARNMT1-dependent cellular methylations yet discovered, as indicated by the dashed line.

Apart from MYLK2, all our identified CARNMT1 substrates are part of C3H ZnFs, suggesting the existence of additional substrates within this group. We observed efficient methylation by CARNMT1 of the MYLK2-derived, 15-mer peptide containing the target histidine, H148, located at the center of the sequence, and we decided to use a similar approach to investigate CARNMT1-mediated methylation of C3H ZnF-derived peptides *in vitro*. In these experiments, individual peptides were synthesized on SPOT peptide arrays, with the MYLK2-derived peptide as a positive control. Peptide arrays were incubated with the recombinant His-tagged Δ52-CARNMT1 and [^3^H]-AdoMet, and methylation was detected by fluorography. We tested the full set of human C3H ZnFs found within ProSite entry PS50103 "Zinc finger C3H1-type profile" (https://prosite.expasy.org/PS50103#TP), representing 119 non-redundant C3H ZnF-derived sequences from 60 different proteins (see Supporting Information 3 for sequences of all tested peptides). Somewhat surprisingly, only a subset of the tested C3H ZnF sequences underwent efficient methylation by CARNMT1 (Fig. 5*A*). These were additionally tested together with the corresponding His-to-Ala mutated peptides as negative controls, confirming that methylation required the presence of the presumed target histidine (Fig. 5*B*). Out of the 119 tested C3H ZnF sequences, 30 were methylated by CARNMT1, and 22 of these were denoted as "strongly" methylated, showing >10% of the methylation level observed with the MYLK2 reference peptide (Fig. 5*C*, Table 1). The strongly methylated sequences encompassed ZFP36L1 (H177), ZFP36L2 (H216), RNF113A (H221), RNF113B (H215), DUS3L (H145), CPSF4L (H59, H86, H114), U2AF1 (H37), MKRN2 (H26, H55, H347), ZPF36 (H128, H166), MKRN1 (H108, H390), CPSF4 (H59), ZC3H15 (H123), DHX57 (H323), ZC3H10 (H96), ZC3H18 (H242), and ZC3H8 (H268). Somewhat surprisingly, H183 in RBM22, which was identified as fully methylated in cells (Fig. 3*C*), did not undergo *in vitro* methylation in the corresponding peptide context. Importantly, 19 (∼86%) of the 22 C3H ZnF sequences reported, by us and others (22, 23), to be methylated in cells were also methylated on peptide arrays (Fig. 5*D*), indicating that *in vitro* methylation represents a good predictor of *in vivo* methylation.

Additionally, we tested 26 non-ZnF sequences, belonging to two categories. The first included sequences reported as histidine-methylated in cells (18), and found in the sequence context F/Y-X-H, which is a common feature of the CARNMT1 target site in MYLK2 and most C3H ZnFs. The second category represented reported non-ZnF substrates of CARNMT1 in cells (22) (see Supporting Information 3 for sequences of all tested peptides). Out of the 26 tested non-ZnF peptides, 10 were methylated by CARNMT1 *in vitro*, but only two of these were strongly methylated (Fig. 5, *B* and *C*), namely, MYLK2 (H148) and MKRN2 (H13). Altogether, we identified 40 peptides that were histidine-methylated by CARNMT1 *in vitro* with variable intensity (Fig. 5, *C*, Tables 1 and 2). Notably, the C3H ZnF peptides generally exhibited considerably higher methylation levels than the non-ZnF peptides. We also investigated whether the peptides that were methylated by CARNMT1 showed preference for particular residues at specific positions (Fig. S4). However, except for the expected preference for F/Y at the −2 position and C at the −4 position (characteristic features of the C3H ZnFs), we did not see any pronounced enrichments. At the remaining positions, several (>10) different amino acids were represented, showing that CARNMT1 can recognize very diverse sequences.

### RNF113A and RNF113B are methylated by CARNMT1 within C3H ZnF domains

**Figure 6:**
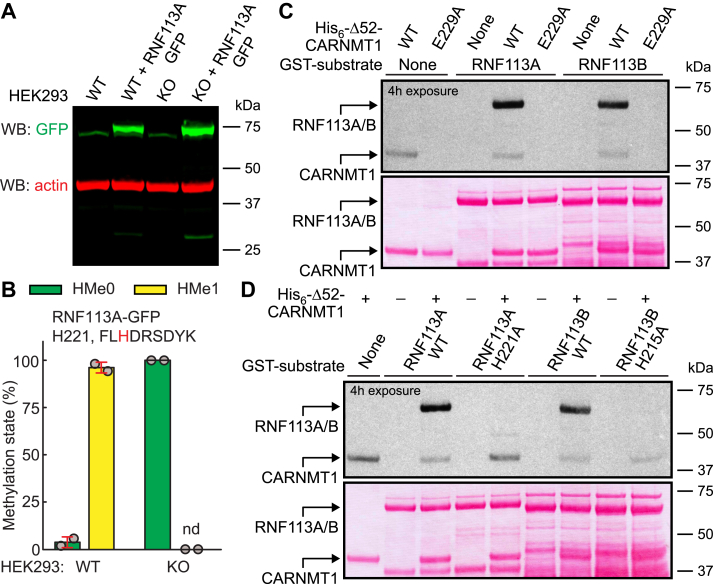
**CARNMT1 methylates RNF113A at H221 in HEK293 TRex cells and *in vitro*.***A*, expression of RNF113A-GFP protein in stably transfected cells. WT and *CARNMT1* KO cells were stably transfected to contain *RNF113A-GFP* gene under control of a doxycycline-inducible promoter. Cells were treated for 24 h with doxycycline (1 μg/ml) to induce expression of RNF113A-GFP, which was detected by western blotting using anti-GFP antibody. Actin is included as a loading control. The non-transfected WT and *CARNMT1* KO cells are included as negative controls. *B*, ectopically expressed RNF113A-GFP is methylated at H221 by endogenous CARNMT1. RNF113A-GFP was expressed in WT or *CARNMT1* KO cells, as in (A). After cell lysis, RNF113A-GFP was immunoprecipitated using GFP-Trap, and separated by SDS-PAGE. The region of the gel containing RNF113A-GFP was cut out, digested with LysC and analyzed by MS. Shown are the mean relative intensities of MS signals, gated for the unmethylated (HMe0) or monomethylated (HMe1) state of the indicated peptide with the targeted histidine marked in *red*. Examples of MS/MS spectra of identified peptides are shown in Supporting Information 2. Error bars represent SD of two independent experiments. nd: not detected. *C*, CARNMT1 methylates recombinant RNF113A and RNF113B. 1 μg of GST-tagged RNF113A or RNF113B was incubated with [^3^H]-AdoMet (0.5 μCi) and 1 μg of His-tagged Δ52-CARNMT1, either WT or E229A-mutated, and then separated by SDS-PAGE and transferred to a membrane. Incorporation of [^3^H]-methyl groups into proteins was visualized by fluorography (*top*) of a Ponceau S-stained membrane (*bottom*). *D*, CARNMT1 methylates RNF113A at H221 and RNF113B at H215, *in vitro*. His-tagged Δ52-CARNMT1 WT was incubated with [^3^H]-AdoMet and GST-tagged RNF113A (WT or H221A-mutated) or RNF113B (WT or H215A-mutated), and then analyzed as in (C). *C, D,* shown are representative images from one of three independent experiments.

Peptide array analysis revealed very efficient methylation of the E3 ubiquitin-protein ligase RING finger protein 113A (RNF113A) at H221, and of the highly similar RNF113B at H215. We attempted to investigate the corresponding methylation events in cells but were hindered by the low abundance of endogenous RNF113 proteins. Instead, we engineered WT and *CARNMT1* KO HEK293 TRex cells ectopically expressing RNF113A-GFP under a doxycycline-inducible promoter (Fig. 6*A*). RNF113A-GFP expressed in doxycycline-induced cells was affinity-purified and subjected to MS analysis, revealing that H221 in RNF113A was ∼95% methylated in WT cells, but completely unmethylated in *CARNMT1* KO cells (Fig. 6*B* and Supporting Information 2 for MS/MS spectra). To establish that RNF113A is directly methylated by CARNMT1, we incubated recombinant GST-tagged RNF113A with His-tagged Δ52-CARNMT1 and [^3^H]-AdoMet. Fluorography confirmed efficient methylation of RNF113A by WT CARNMT1, but not by the E229A mutant (Fig. 6*C*). The highly similar RNF113B paralogue was also efficiently methylated *in vitro* (Fig. 6*C*), and for both RNF113 proteins, mutation of the target histidine (H221A in RNF113A, H215A in RNF113B) completely abolished methylation (Fig. 6*D*). These results demonstrate that both RNF113 paralogs are efficiently methylated by CARNMT1 *in vitro* and provide direct evidence that RNF113A is also efficiently methylated by CARNMT1 in cells.

### Sequence specificity of CARNMT1

The 30 C3H ZnF-derived peptides methylated by CARNMT1 did not share any apparent consensus sequence that distinguished them from the non-methylated peptides (Fig. S4). This suggests that a strict recognition motif may not exist, despite the enzyme's apparent strong selectivity for certain C3H ZnF-derived, His-containing sequences.

**Figure 7:**
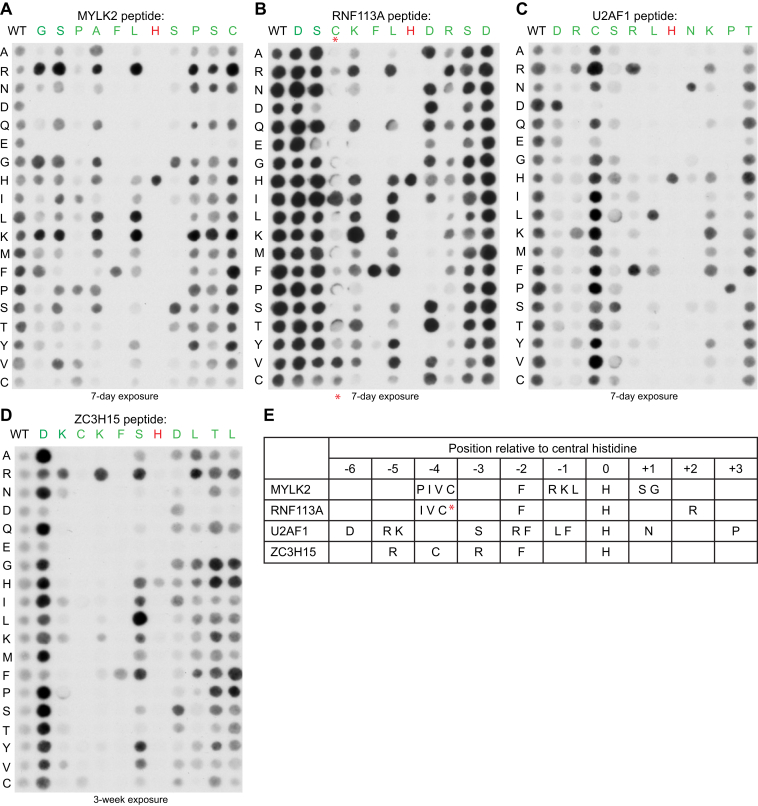
**Different CARNMT1 substrate peptides yield distinct sequence specificity profiles.** SPOT peptide arrays were prepared using 15-mer peptides derived from **(**A) MYLK2 (RGSPAFLHSPSCPAI), (B) RNF113A (GDSCKFLHDRSDYKH), (C) U2AF1 (GDRCSRLHNKPTFSQ), and (D) ZC3H15 (GDKCKFSHDLTLERK). The shown residues (*red* and *green*) in each peptide were individually replaced with all other amino acids, except tryptophan. The arrays were incubated with [^3^H]-AdoMet (0.5 mCi/ml) and His-tagged Δ52-CARNMT1 (0.1 μM). Incorporation of [^3^H]-methyl groups into peptides was visualized by fluorography. Shown are representative images of peptide arrays from one of two independent experiments. See Supporting Information 3 for sequences of all tested peptides. *E*, CARNMT1 has a complex sequence-recognition pattern that depends on the individual peptide substrate. Shown are summarized results of CARNMT1 amino acid preferences at individual positions in the four peptides. *Red* asterisks indicate that the data corresponding to −4 position of RNF113A peptide, in (B), were of poor quality and for this position, the specificities indicated in (E) has been based on a different array shown in Fig. S5.

To further explore this, we systematically replaced the residues surrounding the target histidine in four different peptides, and tested CARNMT1-mediated methylation of the substituted peptides on peptide arrays. These peptides were from the initially discovered MYLK2, which is a non-ZnF sequence, as well as three C3H ZnF peptides derived from RNF113A, U2AF1, and ZC3H15. The results showed a position-specific sequence preference profile for each individual peptide (Fig. 7, *A*–*D*), but the actual residue(s) preferred at a given position varied considerably between the different peptides. Moreover, the degree of selectivity, *i.e.*, the requirement for specific amino acids at a given position, also varied considerably, indicating that sequence recognition by CARNMT1 depends on the overall sequence context of the substrate, rather than a fixed sequence motif. The results of these experiments are summarized in Figure 7*E*, and here, we highlight some examples. Three of the four peptides contain an F residue at the −2 position (relative to the target histidine), which is also a common feature of the C3H ZnFs (Fig. 3*E*), and these showed a near-absolute requirement for an F at this position. In contrast, for the U2AF1-derived peptide, which has an atypical R at this position, both F and R were tolerated. Also, while the U2AF1 peptide showed a strict requirement for a P at +3 position, almost all residues were tolerated at this position in the other three peptides. The +1 position represents the most extreme example of deviating amino acid requirements between the four peptides; S or G are preferred in MYLK2 and N in U2AF1, whereas most residues are tolerated in RNF113A and ZC3H15. Within the C3H ZnFs, a Cys residue at the −4 position is required for Zn coordination, but regarding CARNMT1-mediated methylation, ZC3H15 was the only of the three tested C3H ZnF peptides that strictly required a Cys at this position. Furthermore, while most substitutions resulted in decreased or unaltered methylation, there were also several examples of substitutions that enhanced methylation over the wild-type sequence, often coinciding with altering the peptide's charge. For example, methylation was enhanced when the positive charge of the MYLK2 peptide was increased by introducing R or K, and when the negative charge was removed from the ZC3H15 peptide by replacing D at the −6 position.

Altogether, these experiments further strengthen the notion that CARNMT1 is a promiscuous protein HMT, with a complex mode of sequence recognition that depends on the overall sequence context of the substrate peptide. For a given position (relative to the central His), some of the four test peptides exhibited a high mutational tolerance, while other sequences were more restrictive. Thus, defining a specific CARNMT1 consensus sequence appears unfeasible.

### The biochemical activity and substrates of CARNMT1 are evolutionarily conserved

**Figure 8:**
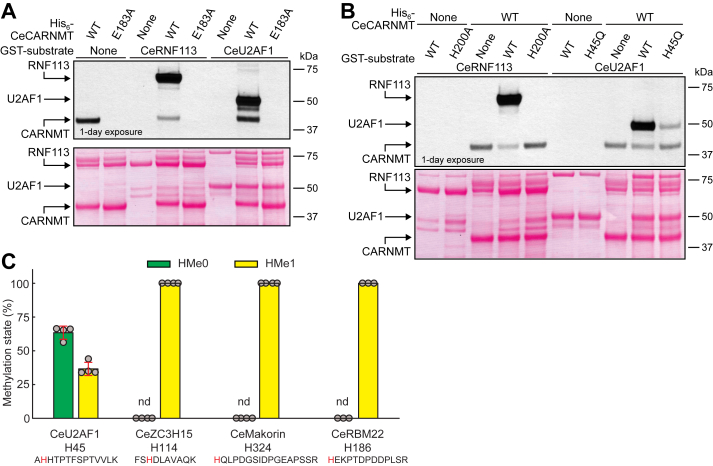
**Methyltransferase activity of CARNMT1 on RNF113A and U2AF1 is evolutionary conserved between human and *C. elegans*.***A***,** recombinant CARNMT from *C. elegans* (Ce) methylates CeRNF113 and CeU2AF1 *in vitro*. 1 μg of GST-tagged CeRNF113 (aa. 1–306) or CeU2AF1 (aa. 1–208) was incubated with [^3^H]-AdoMet (0.5 μCi) and 1 μg His-tagged CeCARNMT, either WT or E183A-mutated, and then separated by SDS-PAGE and transferred to a membrane. Incorporation of [^3^H]-methyl groups into proteins was visualized by fluorography (top) of a Ponceau S-stained membrane (bottom). *B*, nematode CARNMT methylates CeRNF113 at H200 and CeU2AF1 at H45, *in vitro*. His-tagged CeCARNMT was incubated with [^3^H]-AdoMet and GST-tagged CeRNF113 (WT or H200A-mutated) or CeU2AF1 (WT or H45Q-mutated) and then analyzed as in (*A*). *A* and *B*, shown are representative images from one of three independent experiments. *C*, the histidine methylation status of C3H ZnF proteins detected in extracts from *C. elegans*. Nuclear extract, generated from 0.3 ml pellets of *C. elegans* (strain N2), was separated by SDS-PAGE, and two regions of the gel, located between 30 to 40 kDa and between 40 to 50 kDa, were cut out and subjected to trypsin digestion and MS analysis. Shown are the mean relative intensities of MS signals, gated for unmethylated (HMe0) and monomethylated (HMe1) states of the indicated peptides with targeted histidine marked in red. Examples of MS/MS spectra of identified peptides are shown in Supporting Information 2. Error bars represent SD from three to four independent experiments. nd: not detected.

Putative CARNMT1 orthologues are found in a wide range of organisms, and, interestingly, many of the CARNMT1 substrates found in humans are also present in other species. To investigate whether the putative CARNMT1 orthologue from a simple animal, the nematode *C. elegans* (Ce), mediates protein histidine methylation, and whether the actual methylation substrates also are conserved, we expressed the recombinant CeCARNMT (protein Y48E1C.2a), as well as the putative substrates CeRNF113 (protein RNF-113; aa. 1–306) and CeU2AF1 (protein UAF-2; aa. 1–208). Next, we tested the ability of the His-tagged CeCARNMT to methylate the GST-tagged CeRNF113 and CeU2AF1, and indeed, we found that WT CeCARNMT, but not the putatively inactive E183A mutant, methylated both substrates (Fig. 8*A*). Both these substrates have their putative CeCARNMT-targeted histidine located in a single C3H ZnF (H200 in CeRNF113, corresponding to H221 in human RNF113A, and H45 in CeU2AF1, corresponding to H37 in human U2AF1), and mutating these histidines essentially abolished CeCARNMT-mediated methylation (Fig. 8*B*).

To investigate whether histidine methylation at C3H ZnFs also occurs in *C. elegans in vivo*, proteins from nematode nuclear extracts were analyzed by MS. Several histidine-methylated peptides were detected, including C3H ZnF-derived peptides corresponding to CARNMT1 targets observed in humans, *i.e.*, the putative *C. elegans* orthologs of U2AF1 (at H45, also described above), ZC3H15 (F27D4.4; at H114), RBM22 (RBM-22; at H186), and Makorin (LEP-2, at H324) (Fig. 8*C*, Supporting Information 2 for MS/MS spectra). Three of these proteins were fully methylated, whereas the H45 in CeU2AF1 showed ∼40% methylation (Fig. 8*C*). In summary, our results show that CeCARNMT is an active protein HMT that targets many of the same substrates as its human counterpart.

## Discussion

In this report, we identify human CARNMT1 as the protein HMT responsible for the previously reported 1MH modification in MYLK2 (Fig. 1). Using an activity-based approach, combined with protein MS, we further identify a number of CARNMT1 substrates in HEK293-derived cell extracts. This led to the identification of several C3H ZnFs as CARNMT1 substrates that were fully methylated in cells, including previously reported substrates (22, 23), as well as several new ones, such as RBM22, PPP1R10, PRR3, and RNF113A (Figs. 3 and 6). Moreover, we utilized peptide arrays to further explore the substrate repertoire and specificity of CARNMT1. Notably, we found that only 30 out of 119 tested human C3H ZnF-derived peptides were targets of CARNMT1-mediated methylation, 22 of which were strongly methylated (Fig. 5). No obvious sequence features distinguished the methylated from the non-methylated ZnFs (Fig. S4). Moreover, while 10 non-ZnF sequences exhibited methylation by CARNMT1, only 2 were strongly methylated. Systematic amino acid substitutions in four different peptide substrates did not reveal a clear substrate consensus sequence, indicating that CARNMT1 has a complex mode of substrate recognition (Fig. 7). Finally, we show that the activity of CARNMT1 is conserved between humans and the nematode *C. elegans* (Fig. 8).

By incubating recombinant CARNMT1 with cellular extracts in the presence of [^3^H]-AdoMet, we detected several *bona fide* CARNMT1 substrates, *i.e.* proteins that became labeled specifically in the KO extract but not in the WT extract. Conversely, further MS analysis of the extracts revealed that the target histidines in several C3H ZnF proteins were 100% methylated in the WT extract and unmethylated in the KO extract. Some of these high-occupancy methylation events had already been reported (U2AF1, ZC3H15, and ZC3H18), but we also discovered several new ones (RBM22, PPP1R10, and PRR3). By investigating the histidine methylation status of candidate substrates in CARNMT1 overexpressing cells and comparing it with *CARNMT1* KO cells, Shimazu *et al.* identified a number of novel CARNMT1 substrates, which included both C3H ZnFs and non-ZnF sequences (22). However, whereas the majority of the C3H ZnF sequences exhibited high methylation levels, the non-ZnF substrates typically showed lower methylation levels. In that study, particular attention was given to MKRN2, which in cells was found to be subject to CARNMT1-mediated histidine methylation at 10 distinct sites, *i.e.*, on both ZnFs and non-ZnFs. However, non-ZnF methylation was typically very low, unless CARNMT1 was overexpressed, whereas two of the ZnFs were fully methylated even at endogenous CARNMT1 levels (22). The high number of histidine methylation sites within MKRN2 is striking, and, as CARNMT1 exists as a dimer in solution (26), one may speculate that the recruitment of one monomer to a high-affinity C3H ZnF site in MKRN2 may allow methylation of a less optimal, neighboring non-ZnF site by the other monomer. The most prominent non-ZnF CARNMT1 target is the muscle-specific kinase MYLK2, where the relevant sequence is an excellent *in vitro* substrate of CARNMT1, and also was found, nearly 4 decades ago, to be fully methylated in rabbit skeletal muscle (24). However, the low degree of evolutionary conservation of the target histidine (H148 in humans) shows that this methylation event is only restricted to some species. Based on the above, as well as on the consistent detection of high-occupancy C3H ZnF methylations across independent studies (Table 2), we conclude that C3H ZnFs are likely the most biologically relevant targets of CARNMT1.

To further assess the substrate specificity of CARNMT1, we conducted a comprehensive peptide array screen of 119 human C3H ZnF-derived sequences (Fig. 5). Interestingly, we observed methylation of only 30 (∼25%) of the 119 tested sequences. Importantly, 19 of the 30 sequences that scored positive in peptide arrays, had been shown, by us and others (Table 2), to be *in vivo* CARNMT1 substrates. RNF113A, one of the most intensively methylated substrates in the peptide array, was also methylated to high occupancy by CARNMT1 in cells (Fig. 6). Moreover, additional eight of the sequences that were methylated on the peptide arrays, are likely CARNMT1 targets also *in vivo*. These are DHX57 (H323), which was shown to be methylated in cells (18), as well as RNF113B (H215), CPSF4L (H59, H86, H114), ZPF36L2 (H216), ZFP36 (H128), and MKRN1 (H390), which show strong homology to already established *in vivo* substrates of CARNMT1. Thus, 27 (90%) of the 30 sequences that scored positive on peptide array methylation are either confirmed or likely CARNMT1 substrates *in vivo*. In contrast, only three (∼3%) of the 89 sequences that were not methylated on the peptide arrays have been reported to be methylated *in vivo*, *i.e.* RBM22 (H183) (Fig. 5*C*) and CPSF4 (H86, H114) (22). Thus, these results indicate that a peptide sequence methylated on the array is also likely to be methylated *in vivo*, and that only a small subset of human C3H ZnFs are actually *in vivo* substrates of CARNMT1. This strongly suggests that DUS3L (H145), ZC3H10 (H96) and HELZ2 (H243), which we found methylated on peptide arrays, are CARNMT1 substrates also in cells. However, although CARNMT1-mediated methylation on a peptide array appears to be a good predictor of *in vivo* methylation, some sequences were methylated *in vivo*, but not on the array, thus representing “false negatives,” the most prominent example being RBM22 (H183). Most likely, methylation of some substrates may not only require interaction of CARNMT1 with residues in the immediate vicinity of the target histidine, but also with other parts of the substrate protein. Thus, additional CARNMT1 substrates may conceivably be discovered by systematically testing CARNMT1-mediated methylation (*in vitro* or in cells) of full-length C3H ZnF-containing proteins.

To better understand CARNMT1 substrate recognition, we performed systematic amino acid substitutions in four distinct substrate peptides (Fig. 7). Each peptide displayed a rather strict sequence profile, where only one or few amino acids were permitted at several of the positions. However, these profiles, and the actual amino acids tolerated at a given position, varied considerably between the four peptides. This indicates that CARNMT1 has a complex mode of substrate recognition, and a malleable active site, where different substrates are bound in distinct conformations with different residue-specific interactions. Also, our results indicate that it will be challenging to predict novel CARNMT1 substrates based on sequence information of known substrates alone. However, if the CARNMT1 substrate collection were to be substantially expanded in the future, it may be possible to cluster substrate sequences based on their CARNMT1 specificity profiles, thus enabling the prediction of additional substrates.

RNA binding appears to be the primary function of C3H ZnFs, and proteins containing these domains are involved in a wide range of processes related to mRNA, such as splicing and turnover (27). Previous studies have focused on the role of CARNMT1-mediated methylation in RNA splicing, particularly in relation to the key splicing factor and CARNMT1 substrate, U2AF1. It was shown that the His-methylated version of U2AF1 bound more strongly to the 3′ splice site of certain pre-mRNAs, and a number of changes in alternative splicing events were observed in *CARNMT1* KO cells (22, 23). However, we here show that two additional spliceosomal components, RBM22 and RNF113A, which play distinct roles in the complicated process of mRNA splicing (28, 29), also are fully methylated cellular substrates of CARNMT1 (Figs. 3*C* and 6*B*). This suggests that CARNMT1 may exert its effects on mRNA splicing through simultaneous methylation of several C3H ZnF-containing components of the splicing machinery. The effect of CARNMT1-mediated His-methylation on mRNA turnover was studied in the context of the C3H ZnFs of RC3H1 (Roquin) and ZFP36 (TTP), which both bind to specific sequence elements in the 3′UTR of some mRNAs, thus mediating their degradation (22). Interestingly, it was found that His methylation of the C3H ZnFs attenuated mRNA binding of these proteins and, accordingly, mRNA degradation (22). Many C3H ZnFs are stabilized by aromatic π-π stacking between the Zn-coordinating His and a nearby aromatic residue, and Wang *et al.* used molecular dynamics simulations to investigate the effect of His methylation on stacking (23). Interestingly, it was found that methylation enhanced stacking and ZnF stability, but only for those C3H ZnFs that had been found to be methylated (23). It still remains uncertain whether CARNMT1-mediated C3H ZnF methylation plays an active and dynamic role in regulating relevant processes such as RNA splicing and turnover, or whether it constitutively optimizes the performance of the involved components. Of note, almost all the *in vivo* CARNMT1 substrates identified in the present study were completely methylated in cells, suggesting a more static and constitutive role, but more investigations are warranted to clarify this.

Although considerable progress has been made in recent years, the repertoire of biologically relevant substrates of CARNMT1 and the biological function of the CARNMT1-mediated methylation events remain incompletely understood. Human CARNMT1 was initially discovered as being responsible for introducing 1MH into the dipeptide carnosine, thereby generating anserine (20). Both these peptides are abundant and appear to have several functions, *e.g.*, as pH-buffering agents, metal chelators, and antioxidants (21). Thus, future research may address the relative biological contributions of the dipeptide methylation and the recently discovered protein HMT activity of CARNMT1. Of note, several carnosine-lacking organisms, such as the budding yeast, nematode, and fruit fly, still have CARNMT1, suggesting a biologically relevant function other than dipeptide methylation. Correspondingly, we have here found that the protein HMT activity of CARNMT1 is conserved from humans to the nematode *C. elegans*, strongly supporting that protein methylation is the biologically important function of CARNMT1. Interestingly, a third type of function for CARNMT1 has been reported in the fruit fly. It was shown that the putative *Drosophila melanogaster* CARNMT1 ortholog binds AdoMet, but rather than being an MTase, it acts as a sensor of cellular AdoMet levels, thereby regulating mRNA translation in response to AdoMet, and thus methionine, levels (30). In summary, CARNMT1 orthologs appear to fulfill several distinct functions in different organisms, and it will be interesting to explore, through future studies, how these contribute to organismal physiology and evolutionary fitness.

## Materials and methods

### Gene cloning and mutagenesis

The plasmid constructs containing open reading frames (ORFs) and the strategy to generate them are described in detail in Table S1. In brief, ORFs were amplified by PCR using specified cloning primers, and cloned into indicated vectors by ligation-independent cloning using In-Fusion HD Cloning Plus kit (Takara Bio). Mutations within ORFs were introduced by site-directed mutagenesis using PCR splicing by overhang extension (PCR SOEing) using appropriate mutagenic primers, as described previously (31). A similar strategy, involving PCR SOEing, was used to add 3xFLAG-tag to the 3′- end of CARNMT1 ORF. All cloned constructs were Sanger-sequenced.

### Bioinformatics analysis

Sequence alignments were generated using algorithms embedded in the JalView v.2 interface (http://www.jalview.org/). Sequence logos were generated by WebLogo (https://weblogo.berkeley.edu).

### Cell cultures, plasmid transfection, and harvesting

HEK293-derived, Flp-In T-REx 293 (TRex) cells (Thermo Fisher Scientific) were maintained in high-glucose DMEM GlutaMAX medium, supplemented with 10% (v/v) fetal bovine serum and 100 U/ml penicillin-streptomycin (all from Gibco). Cells grown at 50 to 80% confluence were transfected with appropriate plasmids using Lipofectamine 3000 transfection kit (Invitrogen), according to the manufacturer’s instructions. For harvesting, cells grown on plates were washed 2x with PBS, then collected in PBS by scraping and centrifuged (5 min, 450×*g*). The supernatant was carefully removed and the cell pellet used for down-stream applications.

### Generation of *CARNMT1* KO cells

*CARNMT1* KO cells were generated in-house, using CRISPR-Cas9, by transfecting HEK293 TRex cells with pX458-derived plasmids expressing guide RNAs targeting exon 3 or exon 4 of *CARNMT1* gene at positions located upstream of Motif Post I. 48 h after transfection, GFP-positive cells were sorted by FACS and grown for 2 weeks. Next, GFP-negative cells were again sorted by FACS and seeded individually in 96-well plates for colony formation. Frame-shifting events within the *CARNMT1* gene were determined by sequencing genomic DNA from individual clones, using the following sequencing primers for exon3 (CCAGTTGAGGTGAGAGGCTAGTG, CAACTGTAATCTCACACAGAGCATCC) and exon4 (GATTGTGAGAAGTCAGAGTCTTAGC, CACTCTAATAACTAGGCAATTGGTTC). Three different *CARNMT1*-deficient HEK293 TRex cell lines were generated: KO#1 (B4, containing mixture of two *CARNMT1* gene alleles with either 2 bp or 7 bp deletion within exon 4), KO#2 (C7, all *CARNMT1* gene alleles contain identical 1 bp insertion in exon 3), and KO#3 (D7, all *CARNMT1* gene alleles contain identical out-of-frame 6 bp deletion in exon 4, generating a premature in-frame STOP codon). In each case, the introduced mutations generate a premature in-frame STOP codon, resulting in the formation of a truncated CARNMT1 protein that is inactive due to absence of Motif Post I. The sequences of gRNAs and mutations identified in *CARNMT1* KO cells are summarized in Table S2. Unless indicated otherwise, the *CARNMT1* KO cells used in this study refer to KO#1 (B4) cell line.

### Complementation of *CARNMT1* KO cells

WT or E229A-mutated *CARNMT1* gene bearing a C-terminal 3xFLAG-tag was cloned into the pcDNA5/FRT/TO plasmid (Thermo Fisher Scientific) under the control of a doxycycline (Dox)-inducible promoter. Complementation of *CARNMT1* KO HEK293 TRex cells (KO#1, B4 cell line) was done by co-transfection with pcDNA5/FRT/TO-CARNMT1-3xFLAG and pOG44 plasmids, and selection with 200 μg/ml Hygromycin B (Thermo Fisher Scientific). The pool of surviving cells was expanded under Hygromycin B pressure but then maintained in media without antibiotics. To test CARNMT1-3xFLAG expression, cells were induced with 1 μg/ml Dox for 24 h and tested by Western blot for the presence of the FLAG-tag and GAPDH (as loading control) using appropriate antibodies (see Western blot analysis section). For large-scale expression, cells were grown in 15 cm diameter plates until 50 to 80% confluence, then Dox-induced for 24 h to express CARNMT1-3xFLAG, and harvested.

### Generation of stable cell lines expressing RNF113A-GFP

*RNF113A* ORF was first cloned into the pEGFP-N1 plasmid, and then *RNF113A-GFP* fusion ORF was recloned into the pcDNA5/FRT/TO plasmid. HEK293 TRex cells, either WT or *CARNMT1* KO, were then co-transfected with pcDNA5/FRT/TO-RNF113A-GFP and pOG44 plasmids, and selected with 200 μg/ml Hygromycin B. The pool of surviving cells was expanded under Hygromycin B pressure but then maintained in media without antibiotic. To test RNF113A-GFP expression, cells were induced with 1 μg/ml Dox for 24 h and tested by Western blot for the presence of the GFP-tag and actin (as loading control) using appropriate antibodies (see Western blot analysis section). For large-scale expression, cells were grown in 15 cm diameter plates until 50 to 80% confluence, then Dox-induced for 24 h to express RNF113A-GFP, and harvested.

### GFP-Trap immunoprecipitation

Cell pellets of HEK293 TRex cells expressing RNF113A-GFP upon induction with Dox, were lysed at 4 °C, for 10 min, in 0.5 ml of Lysis Buffer (50 mM Tris-HCl, pH 7.4, 100 mM NaCl, 5% glycerol, 1% Triton X-100, supplemented with 1 mM DTT and 2% protease inhibitors cocktail [P8340, Sigma]). The lysate was cleared at 4 °C by centrifugation (10 min, 16,100×*g*) and subjected to immunoprecipitation using magnetic GFP-Trap agarose beads (Chromotek) at 4 °C, for 1 to 2 h. Beads were extensively washed with Lysis Buffer, and immunoprecipitated material was resolved by SDS-PAGE and further processed for mass spectrometry analysis.

### Fractionation of cell extracts

Cells were grown in 15 cm diameter plates until 50 to 80% confluence, harvested and cell pellets were processed further, without freezing. Cell lysis and fractionation into cytoplasmic and nuclear fractions was performed at 4 °C, similarly, as described previously (13). Cell pellets were lysed on ice, for 5 min, in 0.3 ml Fractionation Buffer (PBS supplemented with 0.1% NP-40 and 2% protease inhibitors cocktail [P8340, Sigma]). The lysate was vortexed and 0.1 ml of whole-cell extract (WCE) was transferred to another tube, which was kept on ice. The remaining lysate was centrifuged (45 s, 16,100×*g*) to sediment the nuclei, and 0.1 ml of the supernatant, designated cytoplasmic fraction (Cyt), was transferred to a new tube, which was kept on ice. The rest of the supernatant was discarded, and the nuclear pellet was washed twice in 0.5 ml Lysis Buffer and centrifuged (45 s, 16,100×*g*). After the final centrifugation, the supernatant was discarded, and the nuclear pellet was resuspended in 0.2 ml Fractionation Buffer and designated nuclear fraction (Nucl). WCE, Cyt, and Nucl fractions were adjusted to contain 5 mM CaCl_2_ and 1 U/μl micrococcal nuclease (Thermo Fisher Scientific), then incubated at room temperature (RT) for 6 min, with intensive vortexing every minute, and finally placed on ice. Protein concentration in WCE and fractions was determined using Pierce BCA Protein Assay Kit (Thermo Fisher Scientific). Samples were stored at −80 °C.

### Isolation of nuclei-enriched fraction from *C. elegans*

Pellets of *C. elegans* strain N2 (WT) (∼0.3 ml) were resuspended in 0.3 ml of Fractionation Buffer (PBS, 0.1% NP-40, 2% protease inhibitors cocktail [P8340, Sigma], 2x cOmplete protease inhibitor cocktail (Roche), 1 mM DTT) and homogenized in Dounce homogenizer on ice (tight piston, 150 strokes). The homogenate was pelleted by centrifugation (1 min, 100×*g*, 4 °C). The supernatant containing nuclei was transferred to new 1.5 ml plastic tube kept on ice, while the pellet was resuspended in 0.3 ml Fractionation Buffer and subjected to two more rounds of Dounce homogenization and centrifugation. Supernatants from all rounds of homogenizations were pooled together, centrifuged (2 min, 4 °C, 100×*g*), and the nuclei in the supernatant were harvested by final centrifugation (1 min, 16,100×*g*, 4 °C). The pellet was washed twice with 0.5 ml of Fractionation Buffer and centrifuged (1 min, 16,100×*g*, 4 °C). The final pellet was resuspended in 50 μl Fractionation Buffer supplemented with 5 mM CaCl_2_ and 1 U/μl micrococcal nuclease, and incubated at RT for 6 min, with intensive vortexing every minute. Samples were stored at −80 °C.

### Western blot analysis

Cells grown in 6-well plates were lysed on ice in 0.1 ml Fractionation Buffer (PBS, 0.1% NP-40, 2% protease inhibitors cocktail [P8340, Sigma]). Cell extracts were centrifuged (2 min, 16,100×*g*, 4 °C), and cleared supernatant was separated by SDS-PAGE and transferred to a PVDF Immobilon-FL transfer membrane (Merck), which was stained with Ponceau S and blocked using Odyssey Blocking Buffer (TBS) (Li-Cor) diluted 1:1 (v/v) in Tris Buffer Saline (TBS). The membrane was then incubated with primary antibodies: mouse anti-FLAG (F1804, Sigma-Aldrich, 1:1000), rabbit anti-GAPDH (ab9485, Abcam, 1:1000), rabbit anti-GFP (ab6556, Abcam, 1:1000) or mouse anti-actin (MAB1501, Merck, 1:2000), diluted in Odyssey Blocking Buffer mixed 1:1 (v/v) with TBS, containing 0.05% Tween-20. The primary antibodies were detected with Li-Cor secondary antibodies (1:15,000) coupled with infrared fluorescent dyes, either goat anti-mouse IRDye 680RD, or goat anti-rabbit IRDye 800CW, according to the manufacturer’s instructions, and visualized using LI-COR Odyssey CLx Imaging System. When necessary, the same membrane was re-probed with different primary and secondary antibodies, without stripping. Precision Plus Protein Standards (Bio-Rad), or Cameleon Duo Pre-stained Protein Ladder (Li-Cor), were used to evaluate the size of polypeptides visualized by Western blot.

### Expression and purification of recombinant GST-tagged proteins

The ORF encoding human CARNMT1, or its variants with initial 52 or 79 amino acids deleted (Δ52-and Δ79-CARNMT1), either WT or E229A-mutated, were cloned into the pGEX-6P2 plasmid (GE Healthcare) to contain an N-terminal GST-tag. Similarly, ORF encoding human or *C. elegans* proteins were cloned into pGEX-6P2 plasmid, and the list of generated constructs is provided in Table S1. Plasmids were transformed into *E. coli* strain BL21-CodonPlus (DE3)-RIPL (Agilent) and protein expression was induced overnight with 0.2 mM IPTG, at 18 °C. Isolation of GST-tagged proteins was performed at 4 °C. Bacteria were lysed in Lysis Buffer (50 mM Tris-HCl pH 7.4, 0.5 M NaCl, 1% Triton X-100, 5% glycerol, 1 mM DTT) supplemented with 1x cOmplete (EDTA-free) protease inhibitor cocktail (Roche) and 10 U/ml Benzonase nuclease (Sigma-Aldrich), and sonicated on ice. Lysate was then clarified by centrifugation (30 min, 45,000×*g*). Cleared lysate was applied to column filled with Glutathione Sepharose 4B beads (Merck), equilibrated in Lysis Buffer. The resin was washed sequentially with 15x column volumes (CV) of Wash Buffer 1 (50 mM Tris-HCl pH 7.4, 0.5 M NaCl, 5% glycerol, 1 mM DTT), and then with 5x CV of Wash Buffer 2 (50 mM Tris-HCl pH 7.4, 0.1 M NaCl, 5% glycerol, 1 mM DTT). GST-tagged proteins were eluted in 2x 1 ml of Elution Buffer (50 mM Tris-HCl pH 7.4, 0.1 M NaCl, 5% glycerol, 15 mM reduced glutathione), and buffer-exchanged to Storage Buffer (50 mM Tris-HCl pH 7.4, 0.1 M NaCl, 10% glycerol) using Pierce protein concentrators PES, 10 MWCO (Thermo Fisher Scientific). Proteins were aliquoted and stored at −20 °C. Protein concentration was determined using Pierce BCA Protein Assay Kit, and protein purity was visually assessed by SDS-PAGE and Coomassie Blue staining.

### Expression and purification of recombinant His-tagged proteins

The ORF encoding human CARNMT1 variant with initial 52 amino acids deleted (Δ52 CARNMT1), either WT or E229A-mutated, was cloned into the pET28a plasmid (Novagen) to contain N-terminal 6xHis-tag. Similarly, ORF encoding full-length CARNMT1 homolog from *C. elegans*, either WT or E183A-mutated, was cloned into pET28a plasmid. Plasmids were transformed into *E. coli* strain BL21-CodonPlus (DE3)-RIPL (Agilent) and protein expression was induced overnight with 0.2 mM IPTG at 18 °C. Isolation of 6xHis-tagged proteins was performed at 4 °C. Bacteria were lysed in Lysis Buffer (50 mM Tris-HCl pH 7.4, 0.5 M NaCl, 1% Triton X-100, 5% glycerol, 25 mM imidazole) supplemented with 1x cOmplete (EDTA-free) protease inhibitor cocktail (Roche) and 10 U/ml Benzonase nuclease (Sigma-Aldrich), and sonicated on ice. Lysate was then clarified by centrifugation (30 min, 45,000×*g*). Cleared lysate was applied to column filled with Ni-NTA-agarose beads (Qiagen), equilibrated in Lysis Buffer. The resin was washed sequentially with 10x CV of Wash Buffer (50 mM Tris-HCl pH 7.4, 5% glycerol, 25 mM imidazole) containing 0.5 M NaCl, then with 5x CV of Wash Buffer containing 2.0 M NaCl, then again with 5x CV of Wash Buffer containing 0.5 M NaCl, and finally with 5x CV of Wash Buffer containing 0.1 M NaCl. His-tagged proteins were eluted in 2x 1 ml of Wash Buffer containing 0.1 M NaCl and 0.2 M imidazole, and buffer-exchanged to Storage Buffer (50 mM Tris-HCl pH 7.4, 0.1 M NaCl, 10% glycerol) using Pierce protein concentrators PES, 10 MWCO (Thermo Fisher Scientific). Proteins were aliquoted and stored at −20 °C. Protein concentration was determined using Pierce BCA Protein Assay Kit, and protein purity was visually assessed by SDS-PAGE and Coomassie Blue staining.

### MTase assays using [^3^H]-AdoMet and fluorography

For fluorography analysis of methylation, 10 μl reactions were set up on ice in Storage Buffer (50 mM Tris-HCl pH 7.4, 0.1 M NaCl, 10% glycerol) containing [^3^H]-AdoMet (0.64 μM, 0.5 μCi/reaction, specific activity 78.2 Ci/mmol) (PerkinElmer Inc.). Reactions also contained 1 to 2 μg recombinant CARNMT1 (2–4 μM, GST or 6xHis-tagged, WT or E229A-mutated) and substrate, either ∼1 μg recombinant GST-tagged protein substrate (1–2 μM) or 2 to 5 μg MYLK2-derived synthetic peptide RGSPAFLHSPSCPAI-amide (130–325 μM) (GenScript). When CARNMT1 activity was tested on cellular material, ∼50 μg protein from WCE, Cyt or Nucl fractions was used as substrate. Reaction mixtures were typically incubated at 37 °C, for 1 h. Samples containing recombinant proteins from *C. elegans* were incubated at 25 °C, for 1 h. When indicated, samples containing Nucl fraction were, after incubation, subdivided into soluble and non-soluble parts by centrifugation (5 min, 16,100×*g*). Reaction mixtures were separated by SDS-PAGE, then transferred to PVDF membrane, which was stained with Ponceau S. The membrane was dried, sprayed with Enhance Solution (57.5% v/v 2-methylnaphthalene, 40% v/v pentyl acetate, 2.5% w/v 2,5-diphenyloxazole) and exposed to CL-XPosure Blue X-ray film (Thermo Fisher Scientific) at −80 °C, in the dark, for 1 to 14 days. Films were developed using OPTIMAX X-ray Film Processor (Protec GmbH). Precision Plus Protein Standards (Bio-Rad) was used to evaluate the size of polypeptides after SDS-PAGE, and Glow Writer autoradiography pen (Sigma-Aldrich) was used to mark the position of the standards on PVDF membrane, enabling their visualization by fluorography. All fluorography experiments were performed at least three times, with similar results, and data from representative experiments are shown.

### Peptide SPOT array methylation

Peptide arrays were synthesized on a cellulose membrane using the SPOT synthesis method (32) with a Multipep peptide synthesizer (CEM Corp.) as described (33, 34). The arrays were incubated in Storage Buffer (50 mM Tris pH 7.4, 0.1 M NaCl, 10% glycerol) for 5 min at RT. For methylation, the arrays were incubated with 6xHis Δ52-CARNMT1 (0.1 or 1 μM) and [^3^H]-labelled AdoMet (0.5 mCi/ml; specific activity 82.3 Ci/mmol) (PerkinElmer Inc.) in Storage Buffer for 1 h at RT. Thereafter, the arrays were washed 5 times with buffer (0.1 M NH_4_HCO_3_ and 1% SDS) and incubated for 5 min in ENLIGHTING Rapid Autoradiography Enhancer (PerkinElmer Inc.). To detect methylated substrates the arrays were exposed to Hyperfilm high-performance autoradiography films (GE Healthcare) in the dark at −80 °C for the indicated time. Film development was performed with an Optimus TR developing machine. The signal spot intensity was analyzed in scanned films using the Phoretix Array software (Totallab life science analysis). Each spot intensity was normalized based on the total intensity maximum and minimum of the corresponding array before calculating the average values of at least two replicates.

### MTase assays using unlabeled AdoMet

For mass spectrometry (MS) and amino acid analyses of methylation, 10 μl reactions were set up on ice in Storage Buffer (50 mM Tris-HCl pH 7.4, 0.1 M NaCl, 10% glycerol) containing unlabeled AdoMet (1 mM). To assay CARNMT1-mediated methylation of MYLK2-derived peptide by MS, reactions contained 0.02 μg synthetic peptide RGSPAFLHSPSCPAI-amide (1.3 μM) and 1 μg recombinant 6xHis-Δ52-CARNMT1 (2.3 μM, WT or E229A-mutated). For amino acid analysis, reactions contained 0.2 μg synthetic peptide RGSPAFLHSPSCPAI-amide (13 μM) and 2 μg of recombinant GST-CARNMT1 (2.7 μM, WT or E229A-mutated). Reactions were incubated at 37 °C for 1 h. After incubation, the reaction mixtures were subject to MS analysis, or HCl-hydrolysis followed by amino acid analysis (see the relevant sections below).

### Mass spectrometry analysis of proteins and peptides

Proteins present in indicated cellular fractions, or isolated by GFP-Trap immunoprecipitation, were separated by SDS-PAGE. When indicated, nuclear fraction was subdivided by centrifugation (5 min, 16,100×*g*, 4 °C) into soluble and non-soluble parts, which were individually separated by SDS-PAGE. The gel was stained with Coomassie to visualize protein bands, and the portion of gel containing protein of interest was excised and subject to in-gel proteolytic digestion (37 °C, overnight) using 0.5 μg Trypsin Gold (Promega) or 0.5 μg Lys-C (Roche) per sample. The next day, peptides resulting from in-gel digestion were cleaned by solid-phase extraction (SPE) using a Ziptip-C18 (Millipore).

When indicated, proteins present in the nuclear fraction, or samples containing MYLK2-derived synthetic peptide, were acetone-precipitated and precipitates resuspended in 50 μl 6M urea. Cysteine bonds were reduced by adding 10 mM DTT and incubating at 30 °C for 30 min, then alkylated by adding 25 mM iodoacetamide and incubating at 23 °C for 60 min in the dark. The reaction was quenched by adding 30 mM DTT and incubating at 30 °C for 30 min. The samples were then digested with 4 μg trypsin (37 °C, overnight). The next day the digestion was stopped by adding 1% formic acid and peptides cleaned by SPE using a Ziptip-C18.

Peptides were analyzed by nanoLC-MS using NanoElute-UHPLC coupled to a TimsTOF Pro mass spectrometer (Bruker Daltonics). LC-MS data were analyzed using Peaks Studio X Pro version 10.6 (Bioinformatics Solution Inc.), and searched against in-house maintained human protein sequence database derived from UniProt, using the following search parameters: maximum number of protease miss-cleavages was set to three, parental mass error tolerance was set to 15.0 ppm and fragment mass error tolerance was set to 0.03 Da, whereas methionine oxidation (+15.99 Da) and histidine monomethylation (+14.02 Da) were set as variable modifications. Cysteine propionamidation (+71.04 Da) was set as a variable modification for in-gel digested samples, whereas cysteine carbamidomethylation (+57.02 Da) was set as a fixed modification for samples alkylated with iodoacetamide. During analysis of MYLK2-derived synthetic peptide, amidation of C-terminal isoleucine (−0.98 Da) was set as additional fixed modification. MS/MS spectra of peptides containing the His-monomethylation were manually inspected for presence of modified residues, followed by quantification of His methylation status of peptides using PEAKS Studio. The percentage of methylation was calculated from the ratio between the signal intensity of the methylated peptide, and the total intensity of the peptide, which is the sum of the signals of the methylated + the unmethylated peptide.

### Amino acid analysis by LC-MS/MS

Reaction mixtures containing MYLK2-derived synthetic peptide, were dissolved in 0.3 ml 6 M ultrapure HCl (Thermo Fisher Scientific). HEK293 TRex cells harvested from confluent 15 cm diameter plates were lysed on ice, for 5 min, in 0.5 ml Fractionation Buffer (PBS, supplemented with 0.1% NP-40 and 2% protease inhibitors cocktail [P8340, Sigma]). Proteins present in lysate were then precipitated with TCA (10%) on ice, for 30 min, and then centrifuged (10 min, 16,100×*g*, at 4 °C). Protein pellet was washed with 10% TCA, centrifuged and the supernatant was removed. Protein pellets were dissolved in 0.4 ml 6 M ultrapure HCl (Thermo Fisher Scientific). Samples dissolved in HCl were incubated under vacuum at 120 °C for 48 h, using 1 ml vacuum hydrolysis tubes (Thermo Fisher Scientific). The resulting solutions were transferred to plastic tubes and evaporated at 65 °C overnight. Dry pellets were then dissolved in 2 ml ultrapure H_2_O, the solutions were filtered (0.45 μm) and again evaporated at 65 °C. The remaining dry material was resuspended in 50 μl of 0.1% formic acid and 1MH (πMH) and 3MH (τMH) were quantified using LC-MS/MS. Analytes were separated using an Agilent Infinity II UHPLC system with a Primesep200 column (150 x 2.1 mm ID), with a flow rate of 0.23 ml/min and a gradient of acetonitrile (0–80%) in 0.1% formic acid over 10 min and detected using Agilent 6495 Triple Quadrupole system operating in positive electrospray mode. The following mass transitions were used for detection of analytes: 1MH (170.1–96.0), 3MH (170.1–123.9) and His (175.1–70). All analytes were quantified using appropriate calibration curves (1–500 nM). The content of 1MH and 3MH is expressed as % of total His.

### Statistical analysis

The independent two-sample Student’s *t* test was used to evaluate the probability (*p*-value) that the means of two populations are not different.

## Data availability

The mass spectrometry proteomics data have been deposited to the ProteomeXchange Consortium *via* the PRIDE (PMID: 39494541) partner repository with the dataset identifier PXD060821 and 10.6019/PXD060821.

## Supporting information

This article contains supporting information.

## Conflict of interest

The authors declare that they have no conflicts of interest with the contents of this article.

## References

[1] Petrossian T.C., Clarke S.G. (2011). Uncovering the human methyltransferasome. Mol. Cell Proteomics.

[2] Falnes P.O. (2024). Closing in on human methylation-the versatile family of seven-beta-strand (METTL) methyltransferases. Nucleic. Acids. Res..

[3] Schubert H.L., Blumenthal R.M., Cheng X. (2003). Many paths to methyltransfer: a chronicle of convergence. Trends Biochem. Sci..

[4] Herz H.M., Garruss A., Shilatifard A. (2013). SET for life: biochemical activities and biological functions of SET domain-containing proteins. Trends Biochem. Sci..

[5] Bedford M.T. (2007). Arginine methylation at a glance. J. Cell Sci.

[6] Falnes P.O., Malecki J.M., Herrera M.C., Bengtsen M., Davydova E. (2023). Human seven-beta-strand (METTL) methyltransferases - conquering the universe of protein lysine methylation. J. Biol. Chem..

[7] Figaro S., Scrima N., Buckingham R.H., Heurgue-Hamard V. (2008). HemK2 protein, encoded on human chromosome 21, methylates translation termination factor eRF1. FEBS Lett..

[8] Kwiatkowski S., Drozak J. (2020). Protein histidine methylation. Curr. Protein Pept. Sci..

[9] Moore K.E., Gozani O. (2014). An unexpected journey: lysine methylation across the proteome. Biochim. Biophys. Acta.

[10] Jakobsson M.E. (2021). Enzymology and significance of protein histidine methylation. J. Biol. Chem..

[11] Kwiatkowski S., Seliga A.K., Vertommen D., Terreri M., Ishikawa T., Grabowska I. (2018). SETD3 protein is the actin-specific histidine N-methyltransferase. Elife.

[12] Wilkinson A.W., Diep J., Dai S., Liu S., Ooi Y.S., Song D. (2019). SETD3 is an actin histidine methyltransferase that prevents primary dystocia. Nature.

[13] Malecki J.M., Odonohue M.F., Kim Y., Jakobsson M.E., Gessa L., Pinto R. (2021). Human METTL18 is a histidine-specific methyltransferase that targets RPL3 and affects ribosome biogenesis and function. Nucleic Acids Res..

[14] Matsuura-Suzuki E., Shimazu T., Takahashi M., Kotoshiba K., Suzuki T., Kashiwagi K. (2022). METTL18-mediated histidine methylation of RPL3 modulates translation elongation for proteostasis maintenance. Elife.

[15] Daitoku H., Someya M., Kako K., Hayashi T., Tajima T., Haruki H. (2021). siRNA screening identifies METTL9 as a histidine Npi-methyltransferase that targets the proinflammatory protein S100A9. J. Biol. Chem..

[16] Davydova E., Shimazu T., Schuhmacher M.K., Jakobsson M.E., Willemen H.L.D.M., Liu T. (2021). The methyltransferase METTL9 mediates pervasive 1-methylhistidine modification in mammalian proteomes. Nat. Commun..

[17] Lv M., Cao D., Zhang L., Hu C., Li S., Zhang P. (2021). METTL9 mediated N1-histidine methylation of zinc transporters is required for tumor growth. Protein Cell.

[18] Kapell S., Jakobsson M.E. (2021). Large-scale identification of protein histidine methylation in human cells. NAR Genom Bioinform.

[19] Ning Z., Star A.T., Mierzwa A., Lanouette S., Mayne J., Couture J.F. (2016). A charge-suppressing strategy for probing protein methylation. Chem. Commun. (Camb).

[20] Drozak J., Piecuch M., Poleszak O., Kozlowski P., Chrobok L., Baelde H.J. (2015). UPF0586 protein C9orf41 homolog is anserine-producing methyltransferase. J. Biol. Chem..

[21] Boldyrev A.A., Aldini G., Derave W. (2013). Physiology and pathophysiology of carnosine. Physiol. Rev..

[22] Shimazu T., Yoshimoto R., Kotoshiba K., Suzuki T., Matoba S., Hirose M. (2023). Histidine N1-position-specific methyltransferase CARNMT1 targets C3H zinc finger proteins and modulates RNA metabolism. Genes Dev..

[23] Wang K., Zhang L., Zhang S., Liu Y., Mao J., Liu Z. (2024). Metabolic labeling based methylome profiling enables functional dissection of histidine methylation in C3H1 zinc fingers. Nat. Commun..

[24] Meyer H.E., Mayr G.W. (1987). N pi-methylhistidine in myosin-light-chain kinase. Biol. Chem. Hoppe Seyler.

[25] Falnes P.O., Jakobsson M.E., Davydova E., Ho A.Y., Malecki J. (2016). Protein lysine methylation by seven-β-strand methyltransferases. Biochem. J..

[26] Cao R., Zhang X., Liu X., Li Y., Li H. (2018). Molecular basis for histidine N1 position-specific methylation by CARNMT1. Cell Res.

[27] Hall T.M. (2005). Multiple modes of RNA recognition by zinc finger proteins. Curr. Opin. Struct. Biol..

[28] Rasche N., Dybkov O., Schmitzova J., Akyildiz B., Fabrizio P., Luhrmann R. (2012). Cwc2 and its human homologue RBM22 promote an active conformation of the spliceosome catalytic centre. EMBO J..

[29] Zhang X., Yan C., Zhan X., Li L., Lei J., Shi Y. (2018). Structure of the human activated spliceosome in three conformational states. Cell Res.

[30] Liu G.Y., Jouandin P., Bahng R.E., Perrimon N., Sabatini D.M. (2024). An evolutionary mechanism to assimilate new nutrient sensors into the mTORC1 pathway. Nat. Commun..

[31] Malecki J.M., Willemen H.L.D.M., Pinto R., Ho A.Y.Y., Moen A., Kjonstad I.F. (2018). Lysine methylation by the mitochondrial methyltransferase FAM173B optimizes the function of mitochondrial ATP synthase. J. Biol. Chem..

[32] Frank R. (2002). The SPOT-synthesis technique. Synthetic peptide arrays on membrane supports--principles and applications. J. Immunol. Methods.

[33] Kudithipudi S., Kusevic D., Weirich S., Jeltsch A. (2014). Specificity analysis of protein lysine methyltransferases using SPOT peptide arrays. J. Vis. Exp..

[34] Weirich S., Jeltsch A. (2022). Specificity analysis of protein methyltransferases and discovery of novel substrates using SPOT peptide arrays. Methods Mol. Biol..

