# Supplementary material for: Identification of substrates and sequence requirements for CARNMT1-mediated histidine methylation of C3H zinc fingers
Source: J Biol Chem. 2025 Jun 3;301(7):110335. doi: 10.1016/j.jbc.2025.110335 (PMC12275196; doi:10.1016/j.jbc.2025.110335)
Supplement: Supporting Information 1 [file mmc1.pdf]

SUPPORTING INFORMATION

Identification of substrates and sequence requirements for CARNMT1-mediated histidine methylation of C3H zinc fingers

Jędrzej M. Małecki, Sara Weirich, Manuel Ramirez-Garrastacho, Lars Hagen, Jakin Al-Egly, Jan H. Anonsen, Lisa Schroer, Maria C. Herrera, Erna Davydova, Geir Slupphaug, Albert Jeltsch and Pål Ø. Falnes

LIST OF CONTENTS

Table S1. Summary of cloning strategy and plasmid constructs used in the current study.

Table S2. Summary of gRNAs design and CARNMT1 KO cells used in the current study.

Figure S1. Alignment of CARNMT1 homologs from various organisms.

Figure S2. H148A mutation abolishes CARNMT1-dependent methylation of the N-terminal domain of recombinant MYLK2.

Figure S3. Recombinant CARNMT1 methylates proteins in *CARNMT1* KO HEK293 TRex cell extracts.

Figure S4. Sequence logos of tested sequences.

Figure S5. Sequence specificity of CARNMT1-dependent methylation of C3H ZnF peptide from RNF113A.

ADDITIONAL SUPPORTING INFORMATION

Supporting Information 2. Examples of MS/MS fragmentation spectra of unmethylated and CARNMT1-dependent methylated peptides identified in the current study. (Available as separate Excel file).

Supporting Information 3. List of peptide sequences used in peptide arrays. (Available as separate Excel file).

Table S1. Summary of cloning strategy and plasmid constructs used in the current study.

Construct name	Source of ORF	Restriction sites	Reference sequence	Cloning primers	Mutagenic primers
**pGEX-6P2-CARNMT1**	HEK293 cDNA	BamHI x XhoI	NP_689633.1	fwd:GGGCCCCTGGGATCCATGCAGCGACGGCGTC, rev:ATGCGGCCGCTCGAGTTATTGTGGCTTACGGACCACAAAC	
**pGEX-6P2-Δ52-CARNMT1**	pGEX-6P2-CARNMT1	BamHI x XhoI		fwd:GGGCCCCTGGGATCCATGAGCACCGAGGAGGAGGAGGA, rev:ATGCGGCCGCTCGAGTTATTGTGGCTTACGGACCACAAAC	
**pGEX-6P2-Δ79-CARNMT1**	pGEX-6P2-CARNMT1	BamHI x XhoI		fwd:GGGCCCCTGGGATCCATGCATGAGCGGGTGAACCG, rev:ATGCGGCCGCTCGAGTTATTGTGGCTTACGGACCACAAAC	
**pGEX-6P2-CARNMT1-E229A**	pGEX-6P2-CARNMT1	BamHI x XhoI		fwd:GGGCCCCTGGGATCCATGCAGCGACGGCGTC, rev:ATGCGGCCGCTCGAGTTATTGTGGCTTACGGACCACAAAC	fwd:GCTTGTCAAGGAAATGCATGGAGTTTTTTTATGC, rev:GCATAAAAAAACTCCATGCATTTCCTTGACAAGC
**pET28a-Δ52-CARNMT1**	pGEX-6P2-CARNMT1	NdeI x BamHI		fwd:CGCGGCAGCCATATGAGCACCGAGGAGGAGGAGGAGAGGCT, rev:CTCGAATTCGGATCCTTATTGTGGCTTACGGACCACAAAC	
**pET28a-Δ52-CARNMT1-E229A**	pGEX-6P2-CARNMT1-E229A	NdeI x BamHI		fwd:CGCGGCAGCCATATGAGCACCGAGGAGGAGGAGGAGAGGCT, rev:CTCGAATTCGGATCCTTATTGTGGCTTACGGACCACAAAC	
**pcDNA5-FRT-TO-CARNMT1-3xFLAG**	pGEX-6P2-CARNMT1 and p3xFLAG-CMV14	KpnI x NotI		fwd:CTTAAGCTTGGTACCATGCAGCGACGGCGTCGCCCT, rev:AGACTCGAGCGGCCGCCTACTTGTCATCGTCATCCTTGTAG	fwd:TGTGGTCCGTAAGCCACAAGGATCCCGGGCTGACTAC, rev:GTAGTCAGCCCGGGATCCTTGTGGCTTACGGACCACAAAC
**pcDNA5-FRT-TO-CARNMT1-E229A-3xFLAG**	pGEX-6P2-CARNMT1-E229A and p3xFLAG-CMV14	KpnI x NotI		fwd:CTTAAGCTTGGTACCATGCAGCGACGGCGTCGCCCT, rev:AGACTCGAGCGGCCGCCTACTTGTCATCGTCATCCTTGTAG	fwd:TGTGGTCCGTAAGCCACAAGGATCCCGGGCTGACTAC, rev:GTAGTCAGCCCGGGATCCTTGTGGCTTACGGACCACAAAC

**pET28a-Ce-TEV-CARNMT1**	C.elegans cDNA	NcoI x BamHI	NP_496829.1	fwd1:GGAGATATACCATGCATCATCATCATCATCACAGCAGCGGCCGCGAAAACCTGTATTTTCAGGGC, fwd2:GAAAACCTGTATTTTCAGGGCGAAGAGCAGGAGCCGACGGA rev:CTCGAATTCGGATCCTTATTCATCAGTCTTCTCGGGTTTTC	
**pET28a-Ce-TEV-CARNMT1-E183A**	**pET28a-Ce-TEV-CARNMT1**	NcoI x BamHI		fwd:GGAGATATACCATGCATCATCATCATCATCACAGCAGCGGCCGCGAAAACCTGTATTTTCAGGGC, rev:CTCGAATTCGGATCCTTATTCATCAGTCTTCTCGGGTTTTC	fwd:GATTTACGGTTCAGGGAAATGCGTTCGCATTTTTCATGCTGTTC, rev:GAACAGCATGAAAAATGCGAACGCATTTCCCTGAACCGTAAATC
**pGEX-6P2-MYLK2**	Human skeletal muscle cDNA	BamHI x SalI	NP_149109.1	fwd:GGGCCCCTGGGATCCATGGCGACAGAAAATGGAGCAGTTGA, rev:GCCGCTCGAGTCGACTCAGACCCCCAGAGCCATCAGTG	
**pGEX-6P2-MYLK2-H148A**	pGEX-6P2-MYLK2	BamHI x SalI		fwd:GGGCCCCTGGGATCCATGGCGACAGAAAATGGAGCAGTTGA, rev:GCCGCTCGAGTCGACTCAGACCCCCAGAGCCATCAGTG	fwd:AGGGGCTCACCTGCCTTTCTGGCTAGCCCCAGCTGTCCTGCCATCATCTCCA, rev:AGCCAGAAAGGCAGGTGAGCCCCTCCTGGCTGCTG
**pGEX-6P2-MYLK2-(aa. 1-261)**	pGEX-6P2-MYLK2	BamHI x SalI		fwd:GGGCCCCTGGGATCCATGGCGACAGAAAATGGAGCAGTTGA, rev:GCCGCTCGAGTCGACTCACGGGCAATCATCCAAAATCTGGAAGC	
**pGEX-6P2-MYLK2-(aa. 1-261)-H148A**	pGEX-6P2-MYLK2-H148A	BamHI x SalI		fwd:GGGCCCCTGGGATCCATGGCGACAGAAAATGGAGCAGTTGA, rev:GCCGCTCGAGTCGACTCACGGGCAATCATCCAAAATCTGGAAGC	
**pGEX-6P2-U2AF1**	HEK293 cDNA	BamHI x SalI	NP_006749.1	fwd:GGGCCCCTGGGATCCATGGCGGAGTATCTGGCCTCCA, rev:GCCGCTCGAGTCGACTCAGAATCGCCCAGATCTTTCACGA	
**pGEX-6P2-U2AF1-(aa. 1-199)**	pGEX-6P2-U2AF1	BamHI x SalI		fwd:GGGCCCCTGGGATCCATGGCGGAGTATCTGGCCTCCA, rev:GCCGCTCGAGTCGACTCAGGATCGGGATCTTGATCTATGCTTC	

**pGEX-6P2-U2AF1-(aa. 1-199)-H37Q**	pGEX-6P2-U2AF1	BamHI x SalI		fwd:GGGCCCCTGGGATCCATGGCGGAGTATCTGGCCTCCA, rev:GCCGCTCGAGTCGACTCAGGATCGGGATCTTGATCTATGCTTC	fwd:GGTGCTCTCGGTTGCAGAATAAACCGACGTTTAG, rev:CTAAACGTCGGTTTATTCTGCAACCGAGAGCACC
**pGEX-6P2-Ce-U2AF1-(aa. 1-208)**	C.elegans cDNA	BamHI x SalI		fwd:GGGCCCCTGGGATCCATGTCGTATGGTGACGGGCTC, rev:GCCGCTCGAGTCGACTTAATAATGTCCAGCTGCGTCGGCT	
**pGEX-6P2-Ce-U2AF1-(aa. 1-208)-H45Q**	pGEX-6P2-Ce-U2AF1-(aa. 1-208)	BamHI x SalI		fwd:GGGCCCCTGGGATCCATGTCGTATGGTGACGGGCTC, rev:GCCGCTCGAGTCGACTTAATAATGTCCAGCTGCGTCGGCT	fwd:CAAATGCTCCAGAGCCCAACATACACCGACATTCTC, rev:GAGAATGTCGGTGTATGTTGGGCTCTGGAGCATTTG
**pGEX-6P2-RNF113A**	HEK293 cDNA	BamHI x SalI	NP_008909.1	fwd:GGGCCCCTGGGATCCATGGCAGAGCAGCTTTCTCCAGG, rev:GCCGCTCGAGTCGACCTAAGTAATGGGAATTGCATCCTCATCG	
**pGEX-6P2-RNF113A-H221A**	pGEX-6P2-RNF113A	BamHI x SalI		fwd:GGGCCCCTGGGATCCATGGCAGAGCAGCTTTCTCCAGG, rev:GCCGCTCGAGTCGACCTAAGTAATGGGAATTGCATCCTCATCG	fwd:CAGCTGCAAATTCCTCGCTGACCGTTCAGATTACAA, rev:TTGTAATCTGAACGGTCAGCGAGGAATTTGCAGCTG
**pEGFP-N1-RNF113A**	pGEX-6P2-RNF113A	XhoI x BamHI		fwd:GACTCAGATCTCGAGATGGCAGAGCAGCTTTCTCCAGG, rev:GCGACCGGTGGATCCCGAGTAATGGGAATTGCATCCTCATCG	
**pcDNA5-FRT-TO-RNF113A-GFP**	pEGFP-N1-RNF113A	KpnI x NotI		fwd:CTTAAGCTTGGTACCATGGCAGAGCAGCTTTCTCCAGG, rev:AGACTCGAGCGGCCGCTTACTTGTACAGCTCGTCCATGC	
**pGEX-6P2-RNF113B**	Human sperm cDNA	BamHI x SalI	NP_849192.1	fwd:GGGCCCCTGGGATCCATGGCAGCGCCACCTTCTCC, rev:GCCGCTCGAGTCGACTTATCTCTTTTTTCCTTCTGCAGCCTG	
**pGEX-6P2-RNF113B-H215A**	pGEX-6P2-RNF113B	BamHI x SalI		fwd:GGGCCCCTGGGATCCATGGCAGCGCCACCTTCTCC, rev:GCCGCTCGAGTCGACTTATCTCTTTTTTCCTTCTGCAGCCTG	fwd:ACAGCTGCAAATTCCTCGCCGACCGTTCCGATTAC, rev:GTAATCGGAACGGTCGGCGAGGAATTTGCAGCTGT

**pGEX-6P2-Ce-RNF113-(aa. 1-306)**	C.elegans cDNA	BamHI x SalI	NP_499375.1	fwd:GGGCCCCTGGGATCCATGGATCTCTTCCGAAAACCGA, rev:GCCGCTCGAGTCGACTCATTCTTGCTTCTGTTGTTGCTTCTTC	
**pGEX-6P2-Ce-RNF113-(aa. 1-306)-H200A**	pGEX-6P2-Ce-RNF113-(aa. 1-306)	BamHI x SalI		fwd:GGGCCCCTGGGATCCATGGATCTCTTCCGAAAACCGA, rev:GCCGCTCGAGTCGACTCATTCTTGCTTCTGTTGTTGCTTCTTC	fwd:GATTCGTGTAAATTTGTGGCCGACCGATCGGACTATAAAC, rev:GTTTATAGTCCGATCGGTCGGCCACAAATTTACACGAATC

Table S2. Summary of gRNA design and *CARNMT1* KO cells generated in the current study.

gRNA sequence	Targeted exon	Strand	PAM	KO#	CARNMT1 mutations at mRNA level	CARNMT1 mutants at protein level
**gRNA1:**<br>**TCTCACAAACTGTTTCAGCG**	Exon3	minus	TGG	#2<br>(C7)	1 bp insertion (T) after T485:<br>483 GCTTGAA	MQRRRRPPPPTSRLPEGCGGGGGGSEEVEVQFSAGRWGSAAAV<br>SAAAAAATRSTEEEEERLEREHFWKIINAFRYYGTSMHERVNRTER<br>QFRSLPANQQKLLPQFLLHLDKIRKCIDHNQEILLTIVNDCIHMFENK<br>EYGEDGNGKIMPASTFDMDKLKSTLETVCERLE-
**gRNA2:**<br>**ATGCTAGGTTATGCTTGTCA**	Exon4	plus	AGG	#3<br>(D7)	6 bp deletion after G674:<br>672 TTG~~TCAAGG~~AAA	MQRRRRPPPPTSRLPEGCGGGGGGSEEVEVQFSAGRWGSAAAV<br>SAAAAAATRSTEEEEERLEREHFWKIINAFRYYGTSMHERVNRTER<br>QFRSLPANQQKLLPQFLLHLDKIRKCIDHNQEILLTIVNDCIHMFENK<br>EYGEDGNGKIMPASTFDMDKLKSTLKQFVRDWSETGKAERDACY<br>QPIIKEILKNFPKERWDPSKVNILVPGAGLGRLAWEIAMLGYA-
**gRNA3:**<br>**ACCTAGCATAGCTATTTCCC**	Exon4	minus	AGG	#1<br>(B4)	2 bp deletion after A650:<br>648 GGA~~AA~~TAG	MQRRRRPPPPTSRLPEGCGGGGGGSEEVEVQFSAGRWGSAAAV<br>SAAAAAATRSTEEEEERLEREHFWKIINAFRYYGTSMHERVNRTER<br>QFRSLPANQQKLLPQFLLHLDKIRKCIDHNQEILLTIVNDCIHMFENK<br>EYGEDGNGKIMPASTFDMDKLKSTLKQFVRDWSETGKAERDACY<br>QPIIKEILKNFPKERWDPSKVNILVPGAGLGRLAWDSYARLCLSRK-
					7 bp deletion after G642:<br>640 CTG~~GCCTGGG~~AAA	MQRRRRPPPPTSRLPEGCGGGGGGSEEVEVQFSAGRWGSAAAV<br>SAAAAAATRSTEEEEERLEREHFWKIINAFRYYGTSMHERVNRTER<br>QFRSLPANQQKLLPQFLLHLDKIRKCIDHNQEILLTIVNDCIHMFENK<br>EYGEDGNGKIMPASTFDMDKLKSTLKQFVRDWSETGKAERDACY<br>QPIIKEILKNFPKERWDPSKVNILVPGAGLGRLK-

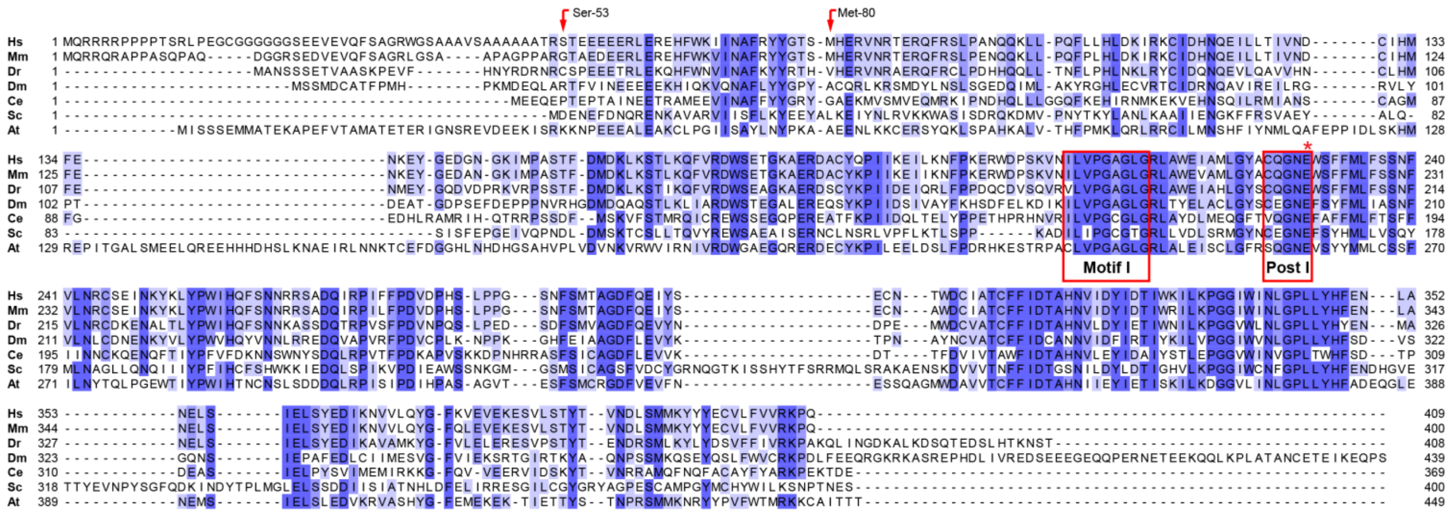

Figure S1. Alignment of CARNMT1 homologs from various organisms. An alignment of CARNMT1 homologs from various organisms was generated applying MUSCLE algorithm embedded in Jalview v.2 on RefSeq sequences of putative CARNMT1 homologs from *Homo sapiens* (Hs, NP_689633.1), *Mus musculus* (Mm, NP_080396.2), *Danio rerio* (Dr, XP_005171825.1), *Drosophila melanogaster* (Dr, NP_569963.1), *Caenorhabditis elegans* (Ce, NP_496829.1), *Saccharomyces cerevisiae* (Sc, NP_014307.1) and *Arabidopsis thaliana* (At, NP_180775.2). The hallmark motifs, Motif I and Post I, found in seven-β-strand MTase members are indicated by red boxes. Red asterisk indicates the position of glutamate located in Motif Post I (i.e. E229 in human CARNMT1) crucial for AdoMet binding. Red arrows indicate the starting amino acid residues, S53 or M80, of recombinant human CARNMT1 variants used in this study, i.e. Δ52- and Δ79-CARNMT1.

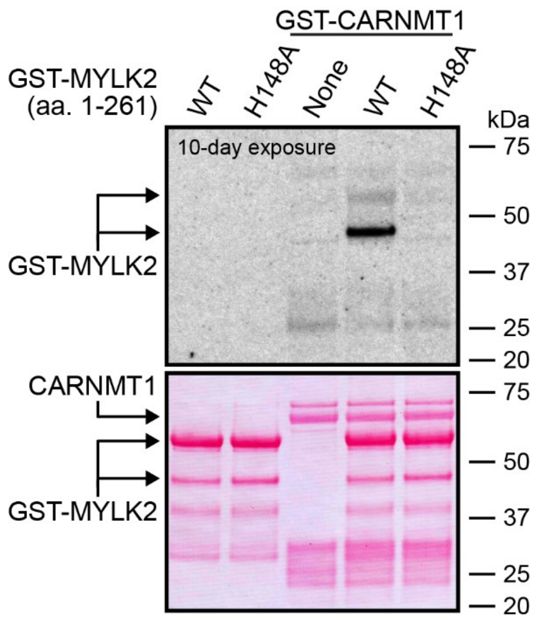

Figure S2. H148A mutation abolishes CARNMT1-dependent methylation of the N-terminal domain of recombinant MYLK2. 1 μg of GST-tagged CARNMT1 was incubated with [^{3}H]-AdoMet (0.5 μCi) and 2 μg of GST-tagged MYLK2 (aa. 1-261), either WT or H148A-mutated, and then analyzed by SDS-PAGE and transferred to a membrane. Incorporation of [^{3}H]-methyl into proteins was visualized by fluorography (top) of a Ponceau S-stained membrane (bottom). Note that GST-MYLK2 (1-261) purifies as a mixture of GST-tagged MYLK2 fragments, with major band of the expected size (53.4 kDa) and a second band located just below 50 kDa and efficiently methylated by CARNMT1, likely representing a C-terminally truncated form. Shown are representative images, from one of three independent experiments.

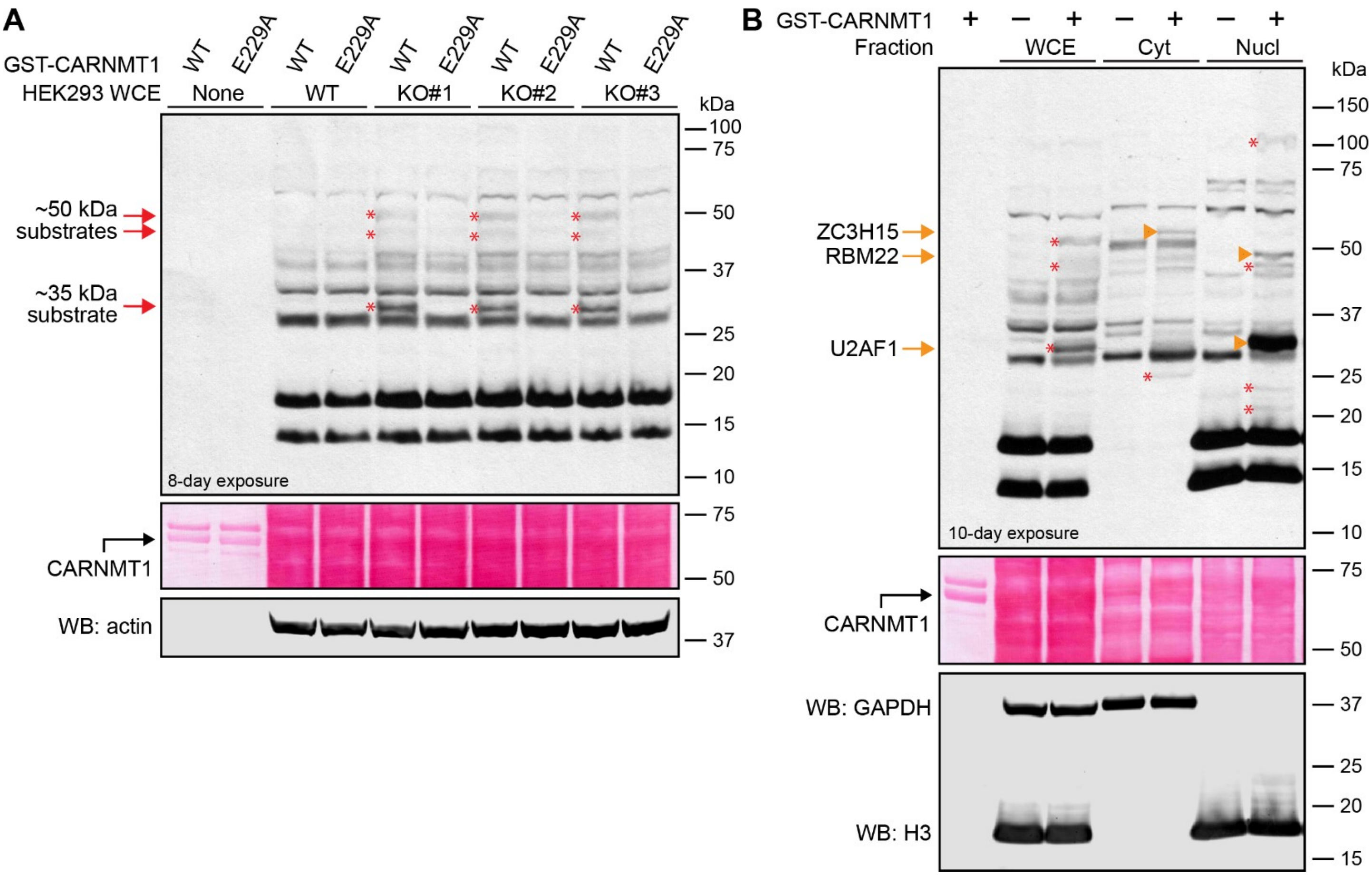

Figure S3. Recombinant CARNMT1 methylates proteins in *CARNMT1* KO HEK293 TRex cell extracts. (A) WT and three different *CARNMT1* KO cells were lysed to generate whole-cell extracts (WCE). 50 μg protein from WCE was incubated with [^{3}H]-AdoMet (0.5 μCi) and 1.5 μg of GST-tagged CARNMT1, either WT or E229A-mutated, and then analyzed by SDS-PAGE and transferred to a membrane. Incorporation of [^{3}H]-methyl into proteins was visualized by fluorography (top) of a Ponceau S-stained membrane (middle). The membrane was probed with anti-actin antibody as a loading control (bottom). The position of [^{3}H]-labeled bands, corresponding to putative CARNMT1 protein substrates, are indicated by red arrows and asterisks. This panel represents an uncropped version of Figure 3A, including the results obtained with three independent KO clones. **(B)** *CARNMT1* KO#1 cells were lysed, and WCE was fractionated into cytosolic (Cyt) and nuclear (Nucl) fractions. 50 μg protein from WCE, or equivalent from Cyt and Nucl fractions, was incubated with [^{3}H]-AdoMet and GST-CARNMT1, and then analyzed as in (A). Expression of glyceraldehyde phosphate dehydrogenase (GAPDH) and histone H3 (H3) are shown as purity and loading controls, for Cyt and Nucl fractions, respectively (bottom). Orange arrows indicate [^{3}H]-labeled bands where U2AF1, ZC3H15 and RBM22 were identified as CARNMT1-dependent histidine-methylated proteins in a parallel MS analysis presented

in Figure 3C. Red asterisks indicated unidentified CARNMT1 substrates. **(A, B)** Shown are representative images from one of three independent experiments.

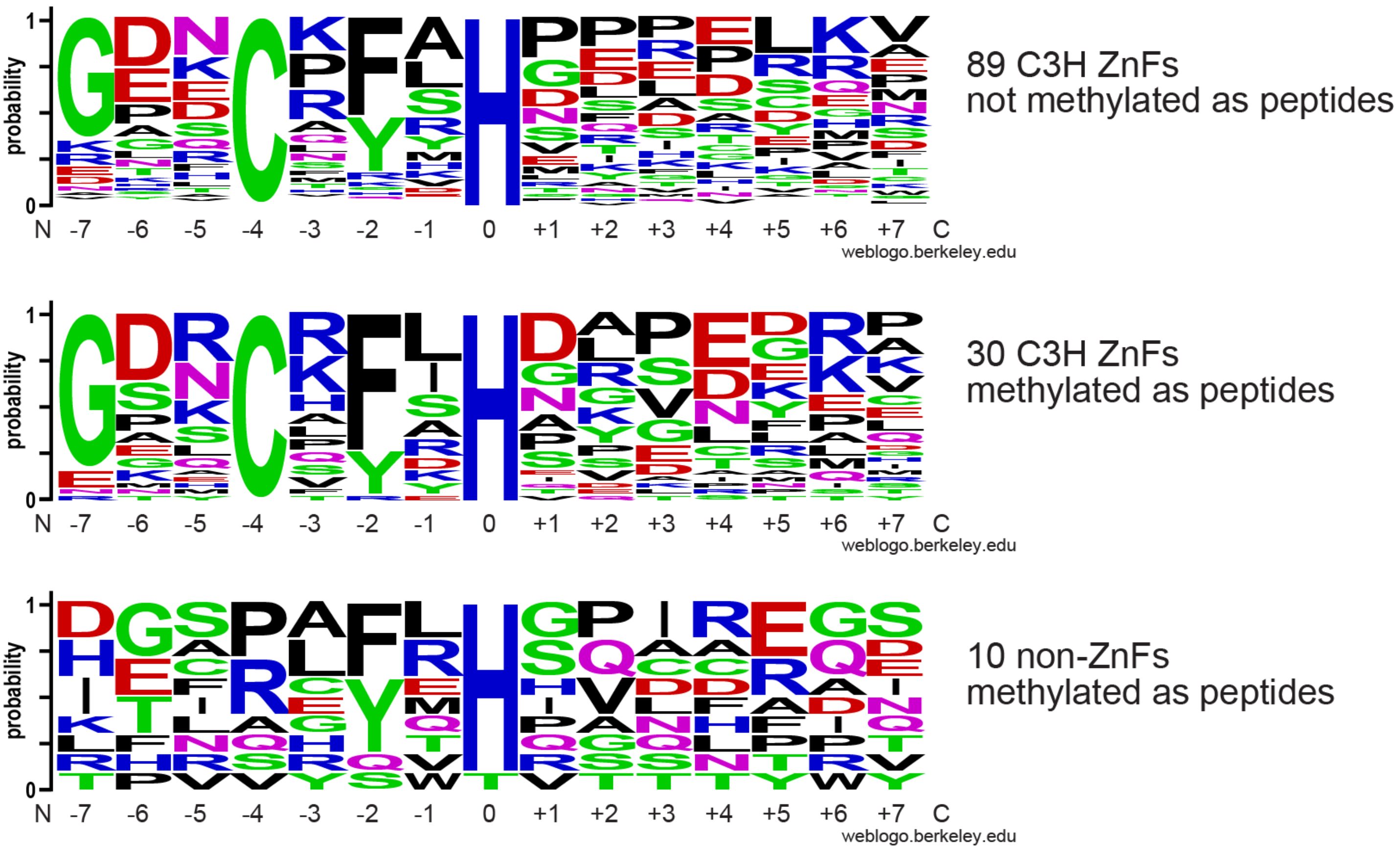

Figure S4. Sequence logos of tested peptides. Sequence logos are expressed as the probability that a given amino acid is present at a particular position within the tested set of sequences. Shown is the sequence logo of 89 tested C3H ZnF peptides that were not methylated by CARNMT1 *in vitro*, and the subset of 30 C3H ZnF peptides that were methylated by CARNMT1 in peptide arrays, as in Figure 5C. Shown is also the sequence logo of 10 non-ZnF sequences that were methylated by CARNMT1, as in Figure 5C.

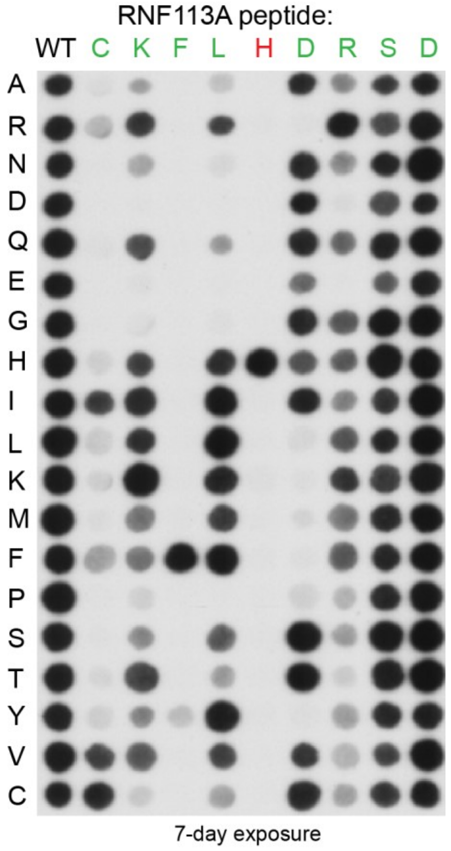

Figure S5. Sequence specificity of CARNMT1-dependent methylation of C3H ZnF peptide from RNF113A. A SPOT peptide array was prepared based on 15-mer peptide, GDSCKFLHDRSDYKH, derived from RNF113A. The shown residues in the peptide (red and green) were individually replaced with all other amino acids, except tryptophan. The array was incubated with [^{3}H]-AdoMet (0.5 µCi/mL; specific activity 82.3 Ci/mmol) and 6xHis Δ52-CARNMT1 (0.1 µM). Incorporation of [^{3}H]-methyl was visualized by autoradiography. Shown is a representative image of peptide array from one of two independent experiments.
